# Blockade of *de novo* dNTP biosynthesis pathway delays HIV-1 early life cycle kinetics and dynamics

**DOI:** 10.1128/mbio.01047-25

**Published:** 2025-06-30

**Authors:** Mohammad Anwar Siddique, Michael D. McRaven, Muhammad Shoaib Arif, Edward J. Allen, Charia McKee, Shravya Honne, Tahmina Sultana, Baek Kim, Ann M. Carias, João I. Mamede, Thomas J. Hope

**Affiliations:** 1 Department of Cell and Developmental Biology, Feinberg School of Medicine, Northwestern University 3270, Chicago, Illinois, USA; 2 Department of Microbial Pathogens and Immunity, Rush University Medical Center 2468, Chicago, Illinois, USA; 3 Center for ViroScience and Cure, Department of Pediatrics, School of Medicine, Emory University 209740, Atlanta, Georgia, USA; The University of North Carolina at Chapel Hill Department of Microbiology and Immunology, Chapel Hill, North Carolina, USA; University of Pittsburgh, Pittsburgh, Pennsylvania, USA

**Keywords:** HIV-1, early life cycle, dNTPs, *de novo* dNTP biosynthesis pathway, ribonucleotide reductase (RNR)

## Abstract

The early events of the HIV-1 life cycle, such as reverse transcription, and capsid shedding commonly known as uncoating, are interdependent and tightly regulated, enabling HIV-1 to adapt to diverse host cells. Here, we explored how host cell dNTP pool size modulates the kinetics and dynamics of HIV-1 reverse transcription and uncoating. We optimized an easy-to-use tool to inhibit the ribonucleotide reductase (RNR) catalyzed *de novo* pathway of dNTP biosynthesis in CHOpgsA-745, HeLa (TZMbl), and owl monkey kidney (OMK) cells. RNR inhibitor rapidly reduced the cellular dNTP pool size, thereby restricting HIV-1 infectivity in a dose-dependent manner. This restriction was reversible upon inhibitor removal, and nucleoside supplementation partially restored infection by enhancing salvage pathways. We find that RNR inhibition slows reverse transcription kinetics and delays the initiation of uncoating in both the capsid integrity and TRIM-CypA restriction assays. Besides, the depletion of intracellular dNTP pools by RNR inhibition leads to significant reductions in both early and late HIV-1 reverse transcription products, with late-stage inhibition comparable to that observed with Nevirapine treatment. To demonstrate the impact of RNR inhibitors on capsid shedding, rather than an off-target effect, we resumed the RNR inhibition-induced delayed initiation of uncoating by reintroducing external dNTPs. This induced recommencement of rapid core integrity loss demonstrating its interplay with the progression of reverse transcription. Therefore, by inhibiting the RNR-catalyzed *de novo* pathway of dNTP biosynthesis, we have reduced the dNTP pool of the host cells to an extent that delays the kinetics and dynamics of HIV-1 early life events.

**IMPORTANCE:**

Cellular dNTP pool homeostasis is maintained by the interplay between the biosynthetic (*de novo* and salvage) pathways and hydrolyzing networks such as SAMHD1. Inhibiting *de novo* pathway using RNR inhibitors reduces the host cell dNTP pool size, thereby restricting HIV-1 infectivity reversibly. Whereas the salvage pathways cannot rescue HIV-1 infectivity to the full extent without the *de novo* pathway. This work correlates HIV-1 infectivity with the dynamic nature of dNTP turnover due to RNR small subunit switching between RRM2 & RRM2B and the action of SAMHD1. The observed modulation of HIV-1 reverse transcription and uncoating in response to RNR inhibition demonstrates the flexibility and adaptability of the virus to replicate in hostile internal cellular environments, which attempt to starve the virus of essential metabolites such as dNTPs. These findings provide insights into how RNR inhibition may impact subsequent steps, such as nuclear localization and integration, offering a foundation for future studies.

## INTRODUCTION

The initial step of HIV-1 infection involves the virus attaching to its specific receptor CD4, and co-receptors CCR5, or CXCR4 on host immune cells. This attachment triggers membrane fusion, enabling the viral core to enter the host cell cytoplasm. Once inside, cytoplasmic deoxynucleotide triphosphates (dNTPs) can diffuse into the intact conical core allowing reverse transcription to convert the viral RNA into double-stranded DNA within a large, dynamic structure often called the reverse transcription complex (RTC) or the viral core (1). The viral core relies on the cell’s microtubule network to be transported through the viscous cytoplasmic environments to the nucleus (1, 2). Upon reaching the nucleus, the viral particle, housing the viral genome, translocates through nuclear pores. The process of reverse transcription can be completed in either the cytoplasm (3) or the nucleus (4). Despite the controversy of different models for uncoating and reverse transcription kinetics and cellular location (5–8), reverse transcription must be completed before integration into the host cell’s DNA, where the provirus becomes a part of the individual cell nuclear host genome (1).

The effectiveness of reverse transcription and subsequent stages of viral replication strongly relies on the quantity and availability of dNTPs within the host cell (9–11). The amounts of dNTPs within a cell are tightly regulated throughout the cell cycle to ensure efficient DNA replication of the approximately 3.2 billion base pairs of the human genome, facilitate mitochondrial DNA maintenance, and minimize excess dNTP accumulation during non-replicative phases to conserve energy and restrict viral replication. Interestingly, HIV target cells, such as resting CD4+ T cells, macrophages, and dendritic cells, maintain low dNTP levels due to the action of the dNTP triphosphohydrolase SAMHD1, which hydrolyzes dNTPs in non-dividing and terminally differentiated cells, thereby restricting HIV-1 reverse transcription (11–16). SAMHD1 is tightly regulated at its expression level, and its enzymatic activity is modulated by phosphorylation (17). The hydrolysis of dNTPs can act as a broad pathogen restriction mechanism preventing replication by starvation for dNTPs. In this way, SAMHD1 limits HIV-1 infection by restricting reverse transcription, leading to reduced viral replication efficiency (11–16). Simian immunodeficiency virus (SIV) or HIV-2 overcomes the limitations imposed by the reduced cellular dNTP pool in these cell types through the accessory protein Vpx which targets SAMHD1 for degradation by the proteasome. The activation of cells can also overcome SAMHD1 activity through specific phosphorylation that inhibits the dNTP hydrolysis activity (9, 11, 13, 18, 19). In addition to the SAMHD1-mediated hydrolysis of dNTPs, another dNTP degradation network orchestrated by 5′-nucleotidases (5′-NT) counters the rate-limiting steps catalyzed by deoxynucleoside kinases in the salvage pathways ensuring the critical level of cellular dNTP pool (20). For those reasons, HIV target cells can have variable levels of dNTPs, and the virus must be able to adapt to successfully infect cells under these different circumstances.

In mammalian cells, dNTPs are synthesized through two primary pathways: *de novo* pathway and salvage pathways. The *de novo* pathway, predominantly active in the cytosol, is responsible for the biosynthesis of most dNTPs, playing a crucial role in maintaining cellular dNTP pools for essential biosynthetic processes, including nuclear DNA replication (20). Conversely, the salvage pathways, which operate in both the cytosol and mitochondria, partially contribute to replenishing the cellular dNTP pool, with a predominant role in supplying dNTPs to the mitochondria (11, 20). The equilibrium of cellular dNTP homeostasis is tightly regulated, ensuring that steady-state dNTP levels can rapidly achieve the levels required for cellular function through the interplay between dNTP biosynthesis and hydrolysis (9, 21).

The key enzyme of the *de novo* pathway, ribonucleotide reductase (RNR) catalyzes the rate-limiting step of reducing ribonucleoside diphosphate to corresponding deoxy-ribonucleoside diphosphate, which is further phosphorylated to generate dNTPs (22–24). RNR is a holoenzyme composed of α-subunit (large subunit) and β-subunit (small subunits), which is believed to attain α_2_β_2_ quaternary state while in its active form (22, 24). The α-subunit encoded by *RRM1* (Ribonucleotide Reductase M1) contains the regulatory site which dictates the overall RNR activity based on its interaction with allosteric effector/inhibitor factors and a specificity site that determines the choice of substrates (22, 24–27). The β-subunit encoded by either *RRM2* or *RRM2B* (also known as p53-inducible RNR small subunit or p53R2) regulates the activity of RNR (28). Both forms of the β-subunit contain a di-nuclear iron center (Fe-O-Fe) and tyrosyl radical which work in concert with α-subunit for the catalytic activity of RNR (22, 24–27). In mammalian cells, the dynamics of RRM2 (regular β-subunit) are tightly regulated at the level of gene expression and protein function/stability and vary with the cell cycle, with peak levels in the S phase. In contrast, RRM2B is constitutively expressed at lower levels and remains stable throughout (29). The reduction reaction mediated by RNR to generate a dNTP requires oxygen. However, the RRM2B subunit can produce low levels of dNTPs needed for the damaged DNA response and for mitochondrial DNA replication (30, 31). Under conditions of reduced O_2_, the RRM2B subunit of RNR combined with dNTPs contributed through the salvage pathway is sufficient to maintain cell life processes without host cell genome replication (30, 31).

This highly dynamic system with multiple pathways of dNTP biosynthesis and catabolism can provide enough dNTPs to rapidly replicate the host cell genome during the S Phase and then quickly reduce the dNTP levels to their minimum. Illustrating the importance of the *de novo* pathway, Bowen et al. (9) reported that Vpx-mediated enhancement of HIV-1 infection in monocyte-derived macrophages (MDMs) requires active dNTP biosynthesis. When RNR was inhibited, Vpx failed to increase dNTP pools despite degrading SAMHD1, demonstrating that *de novo* dNTP biosynthesis is essential for supplying dNTPs necessary for efficient reverse transcription in MDMs. The tight regulation of dNTPs over the cell cycle represents a substantial obstacle for HIV sexual transmission because tissue resident T-cells (Trm) are typically in a G0, quiescent state (32) where dNTP levels are minimal. So even if the virus were to enter the cytoplasm of a Trm in this state, it might not be able to complete reverse transcription and integrate into the host DNA.

Multiple small-molecule inhibitors that disrupt RNR function through different mechanisms can inhibit RNR and the *de novo* pathway in a concentration-dependent manner. The ability of RNR inhibitors to perturb dNTP pools and stress cancer cells toward death has shown great promise in cancer research (22, 25, 33–35). The mechanism of action of small molecule RNR-inhibition can provide further insights. For example, triapine (3-AP) inhibits both RRM2 and RRM2B (36), while RRM2B is less sensitive to hydroxyurea (HU) than RRM2 (37), and COH29 blocks the formation of RNR holoenzymes (33, 35). This association between compromised HIV-1 infectivity and dNTP pool depletion was previously observed in activated peripheral blood lymphocytes (38), suggesting the potential use of HU as an anti-HIV drug (39). In this study, we utilized RNR inhibitors to investigate the impact of limiting dNTP concentrations on reverse transcription and the kinetics and dynamics of the early phase of the HIV-1 life cycle. By examining how limiting dNTP levels influence core uncoating and reverse transcription, we provide further insights into the interdependent processes governing HIV-1 early life events and productive infection.

Although there is much known about the early life cycle of HIV-1, some aspects have sparked considerable debate, with one major point of contention among researchers being the intracellular location where the initiation of the uncoating process occurs. For example, our previous studies have demonstrated HIV-1 uncoating initiates in the cytoplasm in CHOpgsA-745 cells, leading to productive infection, and in primary CD4+ T cells and MDMs, we observed a significant reduction in capsid protein (CA) levels associated with viral complexes upon nuclear localization, compared to levels observed in the cytoplasm and at the nuclear membrane (8, 40). However, other research groups have put forth the nuclear pore (5, 41, 42) or nucleus (4, 7, 43, 44) as the site of initiation and completion of HIV-1 capsid uncoating. Overall, the utilization of diverse model systems and approaches such as different labeling systems has resulted in such disparate outcomes, leaving us without definitive conclusions. Variability in the cellular dNTP pools in different models to study HIV-1 uncoating may influence the variability observed in different systems. Therefore, an investigation into how the cellular dNTP pool influences the early stages of the HIV-1 life cycle will enable us to understand how the host cell intracellular environment dictates the outcomes of critical post-fusion events in the HIV-1 early life cycle.

This study adds an important new tool to extend our established cell-based toolkit that (i) provides insights into drug sensitivity kinetics with a GFP reporter virus infectivity quantified by flow cytometry as readout and (ii) how the process of capsid integrity loss is perturbed utilizing two distinct assays that are cell-based. The first is known as the cyclosporin A (CsA) washout assay (45–47), which can detect the time at which the HIV-1 core has disrupted to a point where it is no longer sensitive to restriction by TRIM-CypA with GFP reporter readout. TRIM-CypA, which restricts HIV infection by targeting the HIV-1 core through the cyclophilin-binding loop of CA, can be reversibly blocked with CsA. As the core disassembles past a certain point, it can no longer be restricted when CSA is washed out. The second method determines the loss of a fluid phase marker within the HIV-1 core during live cell time-lapse fluorescent microscopy. This live cell imaging-based capsid integrity assay identifies the initiation of the shedding of CA allowing GFP to leak out of the core structure in the cytoplasm of living cells (8, 48–51). Altering the dNTP pool by inhibiting *de novo* dNTP biosynthesis with RNR inhibitors or reducing dNTP catabolism with Vpx-targeted degradation of SAMHD1, we will evaluate the impact of this system and can be readily adapted to other assays for early events of the HIV-1 life cycle.

## MATERIALS AND METHODS

### Cells and reagent

The cultivation of 293T, TZMbl, and owl monkey kidney (OMK) cells was carried out in DMEM supplemented with 10% FBS, L-glutamine, and antibiotics, while for CHOpgaA-745 cells, MEM nonessential amino acids were present in addition. All cell lines were maintained at 37°C with 5% CO_2_.

Nevirapine (SML0097, Sigma-Aldrich) was used at a final concentration of 10 µM (CHOpgsA-745 and TZMbl cells) and 5 µM (OMK cells). Cyclosporine/CsA (239835, Sigma-Aldrich) was used at a final concentration of 2.5 µM. Hydroxyurea/HU (H8627, Sigma-Aldrich), COH29 (HY-19931, MedchemExpress), and triapine/3-AP (HY-10082, MedChemExpress) were used at final concentration depending on the cell types based on respective inhibitor dose-response data. Polybrene/hexadimethrine bromide (NC0663391, Fisher Scientific) was used at a final concentration of 5 µg/mL. For dNTP delivery experiments, a phosphate-free tricine buffer was prepared according to the previous report (52).

### Virus production and characterization

The vesicular stomatitis virus G protein (VSV-G) pseudotyped GFP reporter HIV-1 virus preparation was generated through poly(ethylenimine) (PEI) transfection of 293T cells with 6 µg of the HIV-1 proviral plasmid HIV-GFP and 4 µg of the pCMV-VSV-G expression plasmid and characterized by HIV-1 p24 quantification by ELISA and measuring infectivity in CHOpgsA-745 cells as previously described (46).

The VSV-G pseudotyped HIV-1 dual-labeled virus was prepared through PEI transfection of 293T cells, using 3 µg of HIV-Gag-iGFPΔEnv, 4 µg of pCMV-VSV-G, and 0.5-2 μg of OptiGag-mRuby3-Integrase expression plasmids as previously reported (8, 48, 50). Characterization of the VSV-G pseudotyped HIV-1 dual-labeled virus involved measuring infectivity in CHOpgsA-745 cells, labeling efficiency and HIV-1 p24 quantification by ELISA like previously reported methods (8, 48, 50).

Simian immunodeficiency virus (SIV) Vpx virus-like particles (VLP) were prepared by PEI transfection of 293T cells, using 4 µg of SIVmac_251_ and 4 µg of pCMV-VSV-G expression plasmids. The Vpx VLP preparation was characterized by p27 ELISA and SAMHD1 degradation capacity as previously described (11).

### RNR inhibitor dose-response relationship assay

A dose-response relationship assay for RNR inhibitors (HU, 3-AP, and COH29) was conducted by synchronously infecting CHOpgsA-745, TZMbl, and OMK cells with VSV-G pseudotyped HIV-1 GFP reporter virus (dilution that conferred infectivity less than 40% to ensure infectivity is not over saturated) using spinoculation (1,200 × *g*) in the presence of Polybrene at 16°C for 90 min. Following spinoculation, the media was replaced with varying dilutions of HU, 3-AP, or COH29. After 48 h of synchronized infection, cells were harvested and fixed, and the percentage of GFP-positive cells was scored using a BD LSR Fortessa Analyzer.

### RNR inhibitor action reversibility assay

To assess the reversibility of RNR inhibitors’ action, cells were synchronously infected with the HIV-1 GFP reporter virus as previously described. After synchronized infection, cells were incubated for 24 h with the respective RNR inhibitor doses (e.g., CHOpgsA-745 cells: HU-250 µM, COH29-12.50 µM, and TZMbl cells: HU-1000 µM, COH29-35 µM). Following the 24 h incubation, the RNR inhibitors were washed out and replaced with pre-warmed media. After 48 h of synchronized infection, GFP-positive cells were quantified.

### Nucleoside feeding assay

To perform the rescue assay, CHOpgsA-745 cells were fed with nucleosides to support the salvage pathways for dNTP production in the presence of RNR inhibitor pressure. Briefly, cells were synchronously infected with the HIV-1 GFP reporter virus, as described previously. Subsequently, the spinoculation media was replaced with a relatively higher dose of HU (1,000 µM) and COH29 (50 µM), along with varying concentrations (250, 500, and 1,000 µM) of each of the four nucleosides. After 48 h of synchronized infection, GFP-positive cells were quantified.

### dNTP delivery in a cell-based assay

CHOpgsA-745 cells were externally delivered with dNTPs in the presence of RNR inhibitor pressure to assess the rescue of HIV-1 infectivity while inhibiting *de novo* pathway. Briefly, cells were synchronously infected with HIV-1 GFP reporter virus (high amount viruses), as described previously. Subsequently, the spinoculation media was replaced with a relatively higher dose of RNR inhibitor, for example, HU (1,000 µM), and incubated for 2 h. Afterward, dNTPs delivery (once and twice) was performed using a dNTP mixture combined with the BioTracker NTP transporter (SCT064, Sigma-Aldrich) according to the manufacturer’s instruction while maintaining RNR inhibitor pressure. After 48 h of synchronized infection, GFP-positive cells were quantified.

### Cellular dNTP quantification

Cellular hydrophilic metabolome was extracted using methanol (details in supplemental material). Cellular dATPs were measured by HIV-1 RT-based dNTP assay as described (53). Dried dNTP extracts from samples were resuspended in water. A 5′ 32P-end of the 18-nucleotide primer was annealed to 19-nucleotide templates with an A overhang. Reactions containing 2 µL of dNTP extract and the reaction master mix (53) were incubated at 37°C for 5 min. Respectively, 0.5 mM dNTP mix and water were used as positive and negative controls. Ten microliters of 40 mM EDTA and 99% (vol/vol) formamide were added and incubated at 95°C for 5 min. Reactions were viewed on a 14% urea-PAGE gel (National Diagnostics) and analyzed using Amersham Typhoon (Cytiva) within the linear range of the assay, to quantify single-nucleotide extensions of the samples. Besides, dNTP profiling was performed by liquid chromatography–tandem mass spectrometry (LC-MS/MS)-based targeted metabolomics (details in supplemental material).

### Nevirapine addition assay

The impact of host cells treated with RNR inhibitors on the HIV-1 reverse transcription process was assessed by determining the point of its completion as indicated by the loss of sensitivity to the timed addition of nevirapine (NVP) following the previously reported methods (8, 40, 47, 54). Briefly, after synchronized viral infection. CHOpgsA-745, TZMbl, and OMK (in the presence of CsA) cells were treated with varying concentrations of HU and COH29. At various time points, as shown in Fig. 3a, the RNR inhibitor was washed out and replaced with media containing NVP.

### Quantitative PCR for HIV-1 early and late reverse transcription products

qPCR was performed to detect HIV-1 early reverse transcription strong stop DNA products that precede first strand transfer and late reverse transcription products. qPCRs were prepared in triplicate using PR1MA qMAX Green qPCR Mix and HIV-specific primers targeting early reverse transcription (Fw: AACCCACTGCTTAAGCCTCA, Rv: ACCAGAGTCACACAACAGACG) and late reverse transcription (Fw: TGTGTGCCCGTCTGTTGTGT, Rv: CTTCAGCAAGCCGAGTCCTG) products. Primers were used at a final concentration of 400 nM. Standard curves were generated using serial dilutions of HIV and β-actin DNA plasmids containing the target sequences. Amplification was performed on a QuantStudio 5 real-time PCR system with the following cycling conditions: initial denaturation (95°C, 3 min), followed by 40 cycles of denaturation (95°C, 5 s) and annealing-extension (60°C, 30 s). Copy numbers were quantified from standard curves and normalized to cell number using β-actin copy number.

### Cyclosporin A washout assay

We analyzed the HIV-1 core temporal sensitivity to TRIM-CypA in our established cell-based tool-kit system, in OMK cells, following previously established protocols (45–47). OMK cells were preincubated with COH29 (100 µM) for 2 h and then subjected to synchronized infection with HIV-1 GFP reporter virus in the presence of CsA and COH29, as described earlier. Experimental and control conditions were performed as shown in Fig. 4b.

### Live imaging

A DeltaVision wide-field microscope equipped with an EMCCD camera, and environmental control was employed for live imaging as previously described (8, 48–50). Briefly, CHOpgsA-745 cells were seeded in a 35 mm dish embedded with No. 1.5 coverslip (P35G-1.5-14-C, MatTek). Cells were synchronously infected with VSV-G pseudotyped HIV-1 dual-labeled (HIV-1: iGFP and mRuby3-Integrase) virus by incubating media in the presence of 10 mM NH_4_Cl, HU (750 or 1,000 µM) or COH29 (25 µM), polybrene and NucSpot650 nuclear-staining dye for 1 h. After washing out NH_4_Cl and media free-floating viral particles, imaging was performed with a three-minute time-lapse until 2 h in FluoroBrite DMEM supplemented with RNR inhibitor. In control conditions, cells did not receive RNR inhibitor treatment.

### dNTP delivery in live cell imaging assays

External dNTPs were delivered to HU-treated cells during live cell imaging assays using the BioTracker NTP transporter molecule. As described above, the first 2 h of three-minute time-lapse imaging was acquired in the presence of HU (1,000 µM), followed by delivery of unlabeled dNTPs by maintaining HU pressure using a dNTP mixture (2.5 µM each) mixed with the BioTracker NTP transporter molecule (10 µM) in CHOpgsA-745 cells as per manufacturer’s instruction. In a separate control experiment to assess the uptake of dNTP under our live cell experimental setup, the ChomaTide AlexaFluor 594-5-dUTP (10 µM) was delivered to CHOpgsA-745 cells with the transporter molecule (10 µM).

### Live imaging analysis

The acquired time-lapse images were subjected to deconvolution and z-projection using SoftWorx (GE Life Sciences). Subsequently, we tracked the loss of GFP signal within individual mRuby3-Integrase particles over time using Imaris (Bitplane). Mean intensities of GFP and mRuby3 signal were quantified over time using custom Trackpy and Scipy, Scikit-Image Python scripts as previously reported (8, 49, 50). In summary, time series data were tracked and visualized using napari, and mean intensities of the GFP and mRuby3 signals were quantified over time after subtracting background noise.

### Rigor and reproducibility

Each experiment was designed to incorporate a minimum of three technical replicates. For each cell-based experiment conducted, there were at least three biological replicates. Flow cytometry data were analyzed using FlowJo version 10.9. The statistical analysis was performed using Prism 10.2.3.

Scripts are available in Github at https://github.com/joaomamede/Scripts_RNR_Manuscript.

## RESULTS

### Host cells treated with RNR inhibitors reduce HIV-1 infectivity

**Fig 1:**
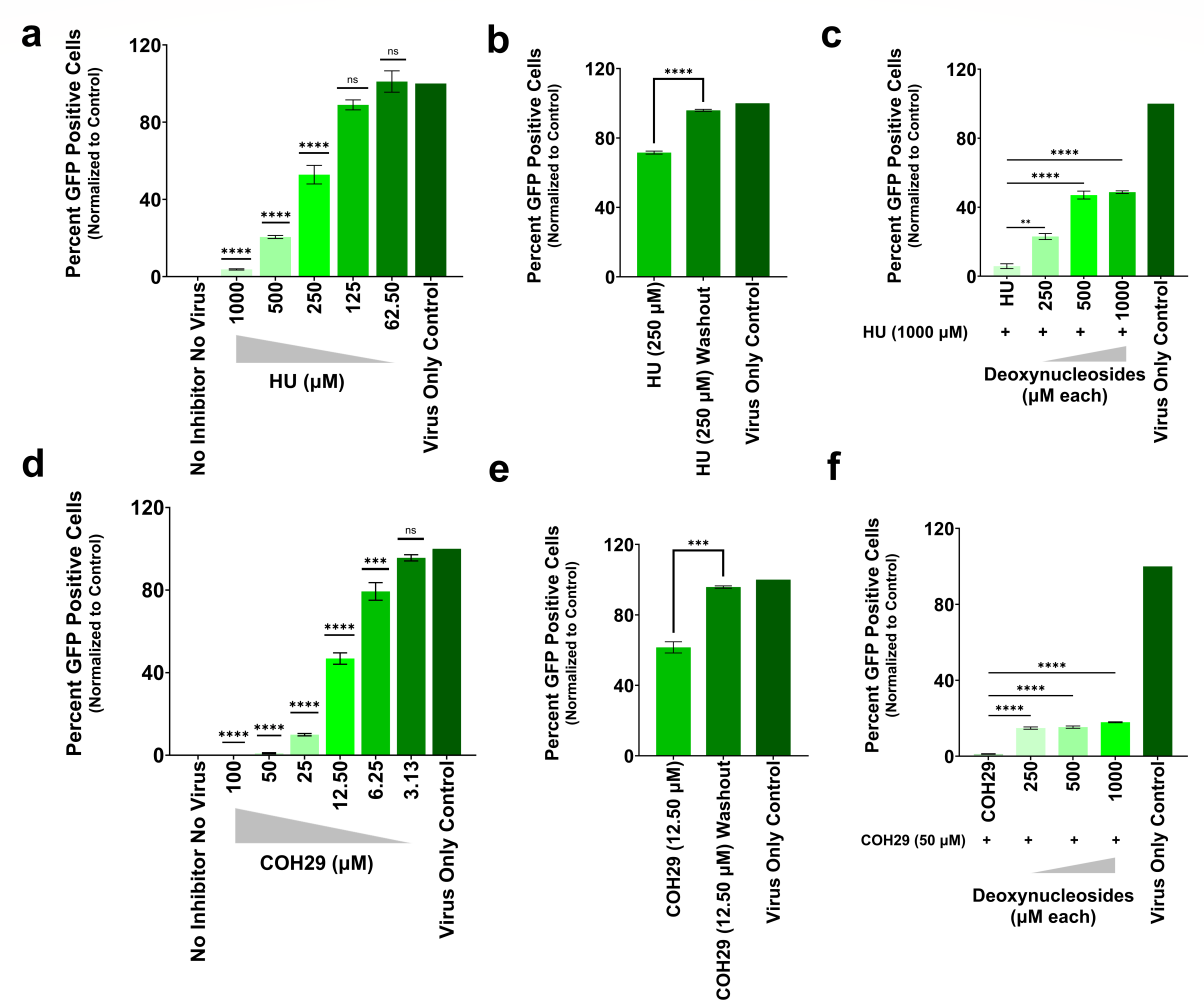
Reduction in HIV-1 infectivity in host cells treated with RNR inhibitors. CHOpgsA-745 cells were synchronously infected with VSV-G pseudotyped GFP reporter HIV-1. (**a, d**) Immediately post-synchronized infection, cells were treated with increasing concentrations of HU and COH29. HIV-1 infectivity was assessed and compared to the virus-only control. (**b, e**) At 24 h post-synchronized infection, HU (250 µM) or COH29 (12.5 µM) treatments were washed out. Subsequent HIV-1 infectivity was compared to continuous RNR inhibitor treatment using an unpaired *t*-test. (**c, f**) Rescue experiments involved exposing CHOpgsA-745 cells to increasing concentrations of nucleosides in the presence of HU (1,000 µM) or COH29 (50 µM). HIV-1 infectivity was then compared to the continuous RNR inhibitor treatment group. In every case, the bar plots shown are the percentage of GFP positive cells at each condition/concentration of inhibitor normalized by setting the percentage of GFP positive cells at virus only control as 100%. Statistical significance was determined using one-way ANOVA with Dunnett’s post-hoc correction to compare each treatment group to the virus-only control. Error bars represent the standard error (SE) from three independent biological replicates. Significance levels are denoted as follows: ****P < 0.0001, ***P = 0.0001, **P = 0.0013, ns = not significant.

We treated cells with RNR inhibitors including HU, 3-AP (RNR β-subunit inhibitor) (55, 56), and COH29 (RNR α- and β-subunit inhibitor) (33, 35). We determined the inhibitor dose-response curve by synchronously infecting cells with VSV-G pseudotyped GFP reporter HIV-1 followed by the replacement of the spinoculation media with media supplemented with RNR inhibitor. We observed a dose-dependent inhibition of HIV-1 infectivity in CHOpgsA-745 cells by culturing in increasing amounts of the RNR inhibitors, HU, 3-AP, and COH29 (Fig. 1a and d; Fig. S1b). We conducted similar experiments in TZMbl cells and HU, 3-AP, and COH29 treatment also significantly reduced HIV-1 infectivity (Fig. S1d through f). At high virus challenge (near saturation of infection), we found that even high concentrations of HU (3,000 µM) were only able to partially inhibit HIV-1 reducing infectivity only to ~40%. Conversely, COH29 and 3-AP were still able to completely restrict HIV-1 infection with the same high titer challenge (Fig. S1c).

We determined the reversibility of RNR inhibitors by washing out both inhibitors (HU or COH29) 24 h after infection in CHOpgsA-745 cells and compared the results with control groups. We used inhibitor concentrations that restricted HIV-1 infectivity to approximately 30%–40% based on RNR inhibitor dose-response data, which are the concentrations planned for use in the reverse transcription kinetics assay. We found that the simple washout of the inhibitors led to the significant and almost complete restoration of HIV-1 infectivity in CHOpgsA-745 cells (Fig. 1b and e). To investigate whether the compromised HIV-1 infectivity was related to the cytotoxic effects induced by the RNR inhibitors, CHOpgsA-745 cells were treated with HU (1,000 µM) or varying concentrations of COH29 (50 µM, 35 µM, and 25 µM) for 24 and 48 h. Cell viability was assessed using GloCell Fixable Viability Dye Red 710 staining, followed by flow cytometry analysis. After 24 h of treatment, cell viability remained unaffected across all treatment conditions. At 48 h, a slight decrease in viability was observed, with less than 7% of cells exhibiting reduced viability compared to untreated controls particularly in the HU and highest COH29 concentration groups (Fig. S1a). Besides, we conducted a rescue experiment exposing the cells to increasing concentrations of nucleosides, which act as precursors for dNTP synthesis through the salvage pathways, while maintaining relatively higher inhibitor pressure. We observed a partial but significant rescue of HIV-1 infection following the introduction of nucleosides (Fig. 1c and f).

### *De novo* pathway blockade by RNR inhibitor causes rapid depletion of host cell dNTP pool

**Fig 2:**
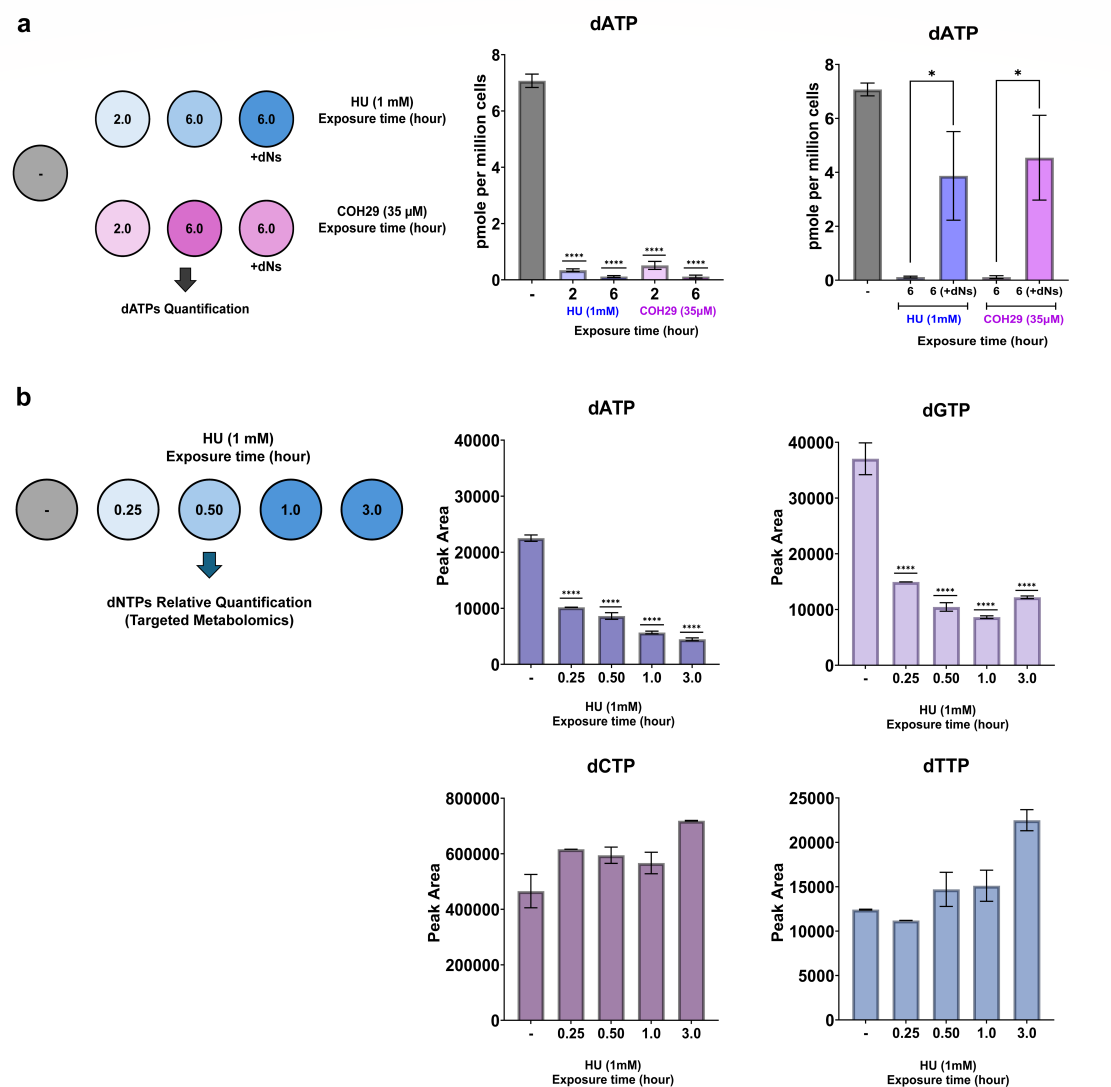
*De novo* pathways blockade by RNR inhibitor causes rapid depletion of host cell dNTP pool. (a) CHOpgsA-745 cells treatment with HU (1,000 µM) or COH29 (35 µM) for 2 and 6 h. After methanol-based metabolite extraction, cellular dATPs were measured by HIV-1 RT-based dNTP assay and comparison with the cellular dATPs level that received no RNR inhibitor treatment. Besides, restoration of cellular dATPs level was measured from CHOpgsA-745 cells with nucleosides (500 µM each) supplementation in the presence of HU (1,000 µM) or COH29 (35 µM) and compared to continuous RNR inhibitor treatment using an unpaired *t*-test. (b) Dynamic turnover of cellular dNTP pool was assessed by CHOpgsA-745 cells treatment with HU (1,000 µM) at different time-points and cellular dNTPs profiling by LC-MS/MS-based targeted metabolomics and comparison with the cellular dNTPs level that received no RNR inhibitor treatment. Statistical significance was determined using one-way ANOVA with Dunnett’s post-hoc correction to compare each treatment group to the no RNR inhibitor treatment control group. For comparisons of dATP restoration following nucleoside supplementation vs RNR inhibitor treatment alone, an unpaired *t*-test was employed. Error bars represent the standard error (SE) from three independent biological replicates. Significance levels are indicated as follows: ****P < 0.0001; *P = 0.0320.

We aimed to investigate the dynamics of host cell dNTP pool depletion when the *de novo* pathway of dNTP biosynthesis is blocked by RNR inhibitors. For this, CHOpgsA-745 cells were treated with either HU or COH29 for different time points as indicated in Fig. 2a. Additionally, cells were supplemented with nucleosides in the presence of RNR inhibitor pressure. Cellular dATPs were measured by HIV-1 RT-based dNTP assay and dNTP profiling by LC-MS/MS-based targeted metabolomics (Fig. 2b).

The HIV-1 RT-based dNTP assay revealed ~90% reduction in cellular dATP levels after 2 h of treatment with either HU or COH29, with a more pronounced decrease observed at 6 h. Nucleoside supplementation significantly restored the dATP pool, achieving 55% and 60% restoration in the presence of HU and COH29, respectively. However, the complete restoration of cellular dNTPs by boosting salvage pathways was not attained, while the *de novo* pathway was inhibited (Fig. 2a).

dNTP profiling by LC-MS/MS-targeted metabolomics indicated a 2.2- and 2.6-fold reduction in the cellular dATP pool at 15 and 30 min post-HU treatment, respectively. After 3 h of HU treatment, a significant 80% reduction in the cellular dATP pool was evident (Fig. 2b). The slight discrepancy in dATP reduction observed between LC-/MSMS metabolomics and the HIV-1 RT-based dNTP assay is likely due to differences in the methods used for quantification, where the lack of certain dNTPs will lead to early and aborted RT products, while the LC-MS/MS metabolomics will represent the detected cellular dNTP pool. Similarly, cellular dGTP levels decreased by approximately 2.5- and 3.5-fold at 15 and 30 min, respectively, following HU treatment which revealed a rapid turnover of purine nucleotide pool in response to the blockade of the *de novo* pathway of dNTP biosynthesis by HU (Fig. 2b). On the other hand, HU-treated CHOpgsA-745 cells indicated minimal or no depletion of pyrimidine nucleotides (dCTP and dTTP) after 3 h of treatment (Fig. 2b). Consistent with previous findings that HU significantly reduces dATP and dGTP pool in CHO cells while increasing dTTP due to its accumulation in the cytoplasm rather than the nucleus (57), we observed a dTTP increase in CHOpgsA-745 cells after 3 h HU treatment.

### Host cells treated with RNR inhibitors compromise HIV-1 reverse transcription kinetics

**Fig 3:**
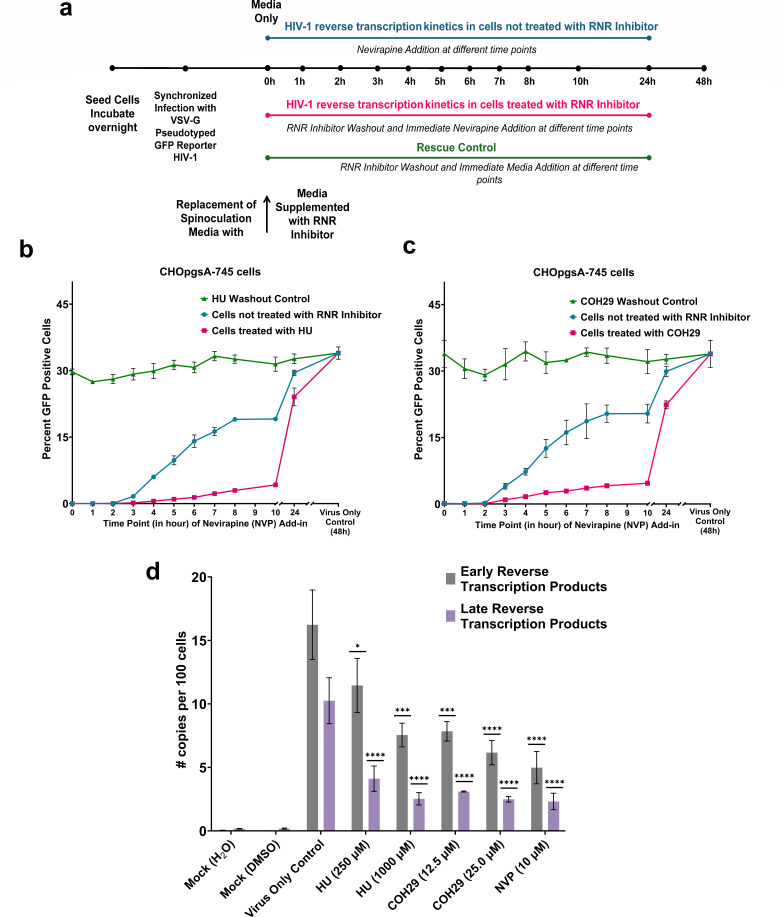
Host cells treated with RNR inhibitors compromise HIV-1 reverse transcription kinetics. (**a**) Experimental layout of the HIV-1 reverse transcription kinetics assay. (**b, c**) Post-synchronized infection, CHOpgsA-745 cells treated with HU (250 µM) or COH29 (12.50 µM) for the duration as indicated in (**a**) followed by washing out and immediately replacing with NVP (10 µM) supplemented media. The sensitivity to NVP treatment (HIV-1 reverse transcription kinetics) was assessed and compared to cells that received no RNR inhibitor treatment. Reversibility of RNR inhibition at each-time point was measured by washing out the inhibitor. Data are representative of three independent biological experiments. Error bars indicate the standard deviation (SD) of three technical replicates within this representative experiment. (**d**) Post-synchronized infection, CHOpgsA-745 cells treated with HU (1,000 µM or 250 µM) or COH29 (25 µM or 12.50 µM) for 7 h followed by comparison of HIV-1 early and late reverse transcription products with virus-only control. Other conditions were mock treatments, NVP (10 µM) treatment. Error bars represent the standard error (SE) from three independent biological repeats. Significance levels are indicated as follows: ****P < 0.0001, ***P = 0.0003, *P = 0.0313.

We investigated how HIV-1 reverse transcription kinetics are affected in cells after RNR inhibitor treatment by determining when infection became resistant to the addition of NVP. CHOpgsA-745 cells were synchronously infected with VSV-G pseudotyped GFP reporter HIV-1. Following spinoculation, the challenge media was replaced with media containing HU (250 µM) or COH29 (12.50 µM). We used HU or COH29 concentrations that resulted in a 30%–40% reduction in HIV-1 infectivity based on RNR inhibitor-dose response data. At different time points, as indicated in Fig. 3a, we washed out the RNR inhibitor by replacing media supplemented with NVP. The experiments were controlled by conditions such as no inhibitors treatment and RNR inhibitor washout at comparable time points.

Our data revealed that the time of sensitivity to NVP was extended in CHOpgsA-745 cell cultures containing 250 µM HU or 12.50 µM COH29 (Fig. 3b and c). In control without RNR inhibitor treatment, it took approximately 6.5 h for 50% of viral particles to become NVP resistant (Fig. S2a). The compromise in the kinetics of HIV-1 reverse transcription in CHOpgsA-745 cells treated with HU or COH29 became evident as early as 3 h post-synchronized infection. The simple removal of inhibitors at similar time points almost entirely restored HIV-1 infection (Fig. 3b and c). However, after 24 h of HU or COH29 treatment in CHOpgsA-745, the cultures had almost caught up to controls with around 80% when compared to HIV-1 reverse transcription completion in control cultures without RNR inhibitor treatment (Fig. S2b).

To further elucidate the effects of RNR inhibition on HIV-1 reverse transcription, we quantified early and late reverse transcription products in CHOpgsA-745 cells under similar treatment conditions. Following synchronized infection and treatment with HU or COH29, cellular DNA was extracted at 7 h post-infection: a time point where 50% of the viral particles had become NVP-resistant. The quantification revealed that low dNTP availability significantly reduced early reverse transcription products. Moreover, late reverse transcription products were reduced to levels comparable to those observed with non-nucleoside reverse transcriptase (RT) inhibitor, NVP (Fig. 3d). In our experimental conditions, we used 10 µM NVP after synchronized infection with VSV-G pseudotyped GFP reporter HIV-1. NVP at this concentration effectively blocks infection (Fig. 3b and c) and significantly reduces the rate and extent of reverse transcription (Fig. 3d). Mechanistic studies show that NVP acts by slowing HIV-1 reverse transcriptase progression rather than fully blocking the enzyme (58). This is consistent with prior reports showing measurable levels of HIV-1 reverse transcription products in cell cultures even after treatment with 5-10 µM NVP (59–61), especially strong-stop DNA. This suggests that dNTP depletion because of RNR inhibition indirectly hampers reverse transcription to a similar extent as RT inhibitor antiretrovirals.

Similar delays in the kinetics of the completion of HIV-1 reverse transcription were observed in TZMbl cells after treatment with 1,000 µM HU or 35 µM COH29 (Fig. S2c and d). In control cultures without RNR inhibitor treatment in TZMbl cells, it took approximately 6.5 h for 50% of viral particles to complete reverse transcription (Fig. S2a). Similarly, the removal of inhibitors at corresponding time points almost entirely restored HIV-1 infection in TZMbl cells (Fig. S2c and d).

### Redistribution of endogenous SAMHD1 after RNR inhibition

Considering the differences in the kinetics of HIV-1 reverse transcription in cells treated with HU or COH29 became evident as early as 3 h post-synchronized infection, we became curious about the dynamics of SAMHD1 expression when the *de novo* pathway is blocked.

To address this, we conducted immunostaining for SAMHD1 to evaluate the amount and localization of SAMHD1 in the cells under investigation. We also performed SAMHD1 staining in THP-1 cells and observed substantial SAMHD1 nuclear expressions (Fig. S3a). We observed that CHOpgsA-745 and OMK cells had lower levels of SAMHD1 compared to unactivated THP-1 cells. Conversely, TZMbl cells exhibited relatively higher levels of SAMHD1 compared to CHOpgsA-745 and OMK cells (Fig. S3a). Furthermore, we noted speckle-like SAMHD1 nuclear signals in these cells. Interestingly, overnight treatment of the cells with HU induced a redistribution of the SAMHD1 from a small number of nuclear speckles to very small puncta that are spread throughout the nucleus (Fig. S3b).

One consequence of drastically reducing dNTP pools with RNR inhibitors is that it will block host cell DNA replication which could be impacting SAMHD1 localization. Next, to explore the relationship between RNR inhibition and changes in SAMHD1 nuclear redistribution, we treated CHOpgsA-745 cells with HU for durations of 3 h, 6 h, and overnight. As a control, we treated cells with Aphidicolin to arrest cell division. We stained the cells for SAMHD1 (Fig. S3b) and quantified the number of SAMHD1 nuclear speckles-like signals or spots, SAMHD1 nuclear signal intensity sum, and the mean intensity of the SAMHD1 nuclear signal (details in supplemental material). We observed a significant increase in the distribution of the number of SAMHD1 nuclear spots in cells treated with overnight HU treatment, while there was no significant increase in distribution in cells treated with HU for 3 h and 6 h (Fig. S4a). Aphidicolin-treated cells also exhibited a similar but more pronounced increase in the number of SAMHD1 nuclear spots (Fig. S4a). Similarly, cells treated overnight with HU exhibited a statistically significant increase in both total and mean SAMHD1 nuclear signal intensity, indicating enhanced nuclear localization and/or redistribution of SAMHD1 in response to *de novo* dNTP synthesis blockade (Fig. S4b and c).

It has been reported that the degradation of SAMHD1 mediated by Vpx nuclear alleles (SIVmac_251_) necessitates the presence of SAMHD1 in the nucleus of the cell (19). Here, we observed that SAMHD1 redistributes within the nucleus of CHOpgsA-745 cells after overnight HU treatment. To ascertain the functional contribution of SAMHD1 in hydrolyzing dNTPs in CHOpgsA-745 cells while blocking the RNR-catalyzed *de novo* pathway of dNTP biosynthesis, we treated CHOpgsA-745 cells with HU overnight while simultaneously administering Vpx (SIVmac_251_ VLP) as previously reported (11). We determined HIV-1 reverse transcription kinetics in HU-treated cells subjected to Vpx VLP treatment or not (Fig. S4d). In cells treated with Vpx VLPs, the progression of HIV-1 reverse transcription kinetics was comparatively faster than in cells without Vpx VLPs treatment, which underscores the functional role of the redistributed endogenous SAMHD1 in hydrolyzing dNTP and HIV-1 restriction during the reverse transcription phase after RNR inhibition (Fig. S4e).

### Host cells treated with RNR inhibitor increase temporal HIV-1 core sensitivity to TRIM-CypA restriction

**Fig 4:**
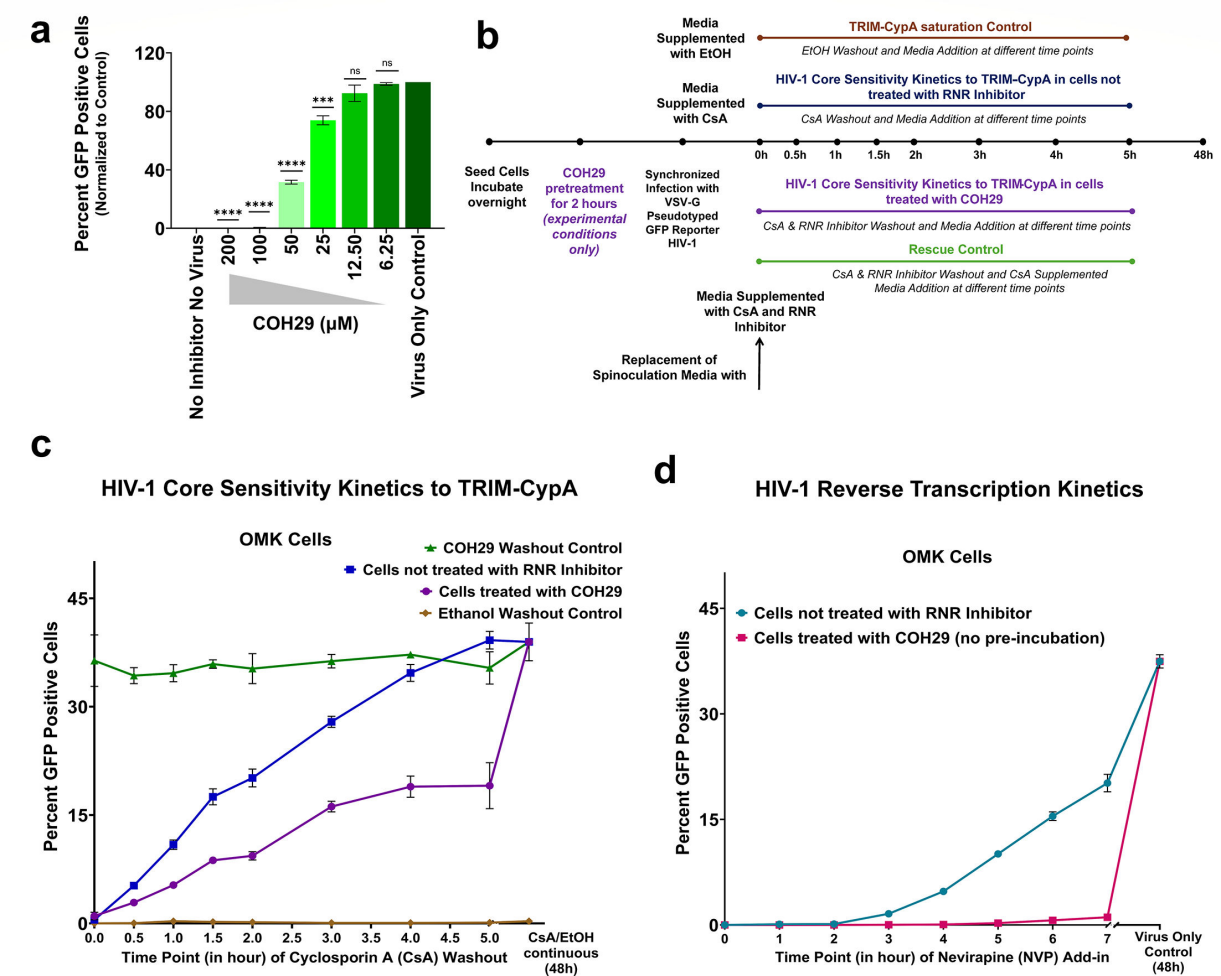
Host cells treated with RNR inhibitor increase temporal HIV-1 core sensitivity to TRIM-CypA restriction. (**a**) OMK cells were synchronously infected with VSV-G pseudotyped GFP reporter HIV-1. Immediately post-synchronized infection, cells were treated with increasing concentrations of COH29 in the presence of CsA (2.5 µM). HIV-1 infectivity was assessed and compared to the virus-only control. The bar plot shown is the percentage of GFP positive cells at each condition/concentration of inhibitor normalized by setting the percentage of GFP positive cells at virus only control as 100%. Error bars represent the standard error (SE) from three independent biological replicates. Significance levels are denoted as follows: ****P < 0.0001, ***P = 0.0002, ns = non-significant. (**b**) Experimental layout to determine the kinetics of HIV-1 core temporal sensitivity to TRIM-CypA. (**c**) OMK cells were pre-incubated with COH29 (100 µM) for 2 h, followed by post-synchronized infection and treatment with COH29 (100 µM) and CsA (2.5 µM) for the indicated durations in (**b**). After treatment, both compounds were washed out and replaced with regular media. HIV-1 capsid core sensitivity to TRIM-CypA was assessed and compared to untreated cells. Additional conditions examined the rescue of HIV-1 infectivity at similar time points and TRIM-CypA restriction functionality after ethanol washout. (**d**) HIV-1 reverse transcription kinetics in OMK cells treated with COH29 (100 µM, no pre-incubation) in the presence of CsA was assessed and compared to cells receiving no COH29 treatment. By comparing HIV-1 core sensitivity to TRIM-CypA and reverse transcription kinetics, the data indicate that HIV-1 cores develop resistance to TRIM-CypA before reverse transcription is completed. Presented curves are representative of three independent biological experiments. Error bars indicate the standard deviation (SD) of three technical replicates within this representative experiment.

After observing a significant compromise in HIV-1 reverse transcription kinetics in cells treated with HU or COH29, our subsequent investigation focuses on understanding the impact of RNR inhibitor treatment on HIV-1 capsid core integrity and uncoating kinetics. Our lab has previously reported a method to measure the kinetics of HIV-1 core susceptibility to TRIM-CypA in OMK cells that uses the washout of the reversible TRIM-CypA binder CsA (46, 47, 54). Initially, we determined the dilution of HIV-1 in OMK cells that resulted in approximately 30%–40% of GFP-positive cells not to affect TRIM-CypA restriction by saturation according to a previous report (45). Next, we investigated the inhibitor-response curve of COH29 in OMK cells and found a significant and gradual reduction in HIV-1 infectivity as the inhibitor concentration increased (Fig. 4a). We also conducted similar experiments with HU in OMK cells, yielding comparable results.

We investigated how HIV-1 sensitivity to TRIM-CypA is affected in OMK cells treated with HU or COH29. However, COH29, which inhibits both α- and β subunits of RNR, showed clearer results. The preincubation of cells with COH29 for ~2 h led to rapid changes in HIV-1 TRIM-CypA sensitivity. To carry out the experiment, we synchronously infected cells with VSV-G pseudotyped GFP reporter HIV-1 in the presence of CsA and COH29. As described in Fig. 4b, we replaced the spinoculation media with media supplemented with COH29 and CsA and washed it out over time by replacing it with regular media to determine the timepoints where TRIM-CypA no longer restricts HIV. We determined HIV-1 core sensitivity to TRIM-CypA in normal cellular conditions, a rescue control (COH29 washout), and TRIM-CypA saturation control (ethanol washout) in OMK cells under our experimental conditions.

In COH29-treated OMK cells, we observed an increase in temporal susceptibility of HIV-1 to restriction by TRIM-CypA after CsA washout compared to cells not exposed to COH29 treatment. The simple removal of COH29 at similar time points almost entirely restored HIV-1 infection. Additionally, TRIM-CypA saturation control demonstrated that TRIM-CypA restriction in OMK cells remained functional (Fig. 4c). Next, we extended our study to determine the HIV-1 reverse transcription kinetics in OMK cells like previously performed in other cells but here in the continuous presence of CsA and observed a similar compromise in HIV-1 reverse transcription kinetics in COH29-treated OMK cells compared to cells that received no COH29 treatment (Fig. 4d). By comparing the kinetics of HIV-1 core sensitivity to TRIM-CypA and reverse transcription kinetics in OMK cells (for example at 2 h post-synchronized infection), we observed that HIV-1 cores become resistant to TRIM-CypA before the completion of reverse transcription suggesting a capsid rearrangement or CA shedding before the completion of reverse transcription. This aligns with the previous findings that capsid remodeling is crucial for successful HIV-1 nuclear entry (6).

### CHOpgsA-745 cells treated with RNR inhibitors delay HIV-1 core initiation of uncoating

The increased sensitivity of HIV-1 cores to the restriction factor TRIM-CypA in COH29-treated OMK cells prompted us to investigate the dynamics of the initiation of HIV-1 uncoating using a more direct microscopy-based assay. Our laboratory has previously developed and validated a Capsid Integrity Assay (8). The assay monitors the levels of GFP as a fluid phase marker within the virion, which also has a second label associated, for example, with Integrase/IN. The GFP, initially located between MA and CA in the Gag polyprotein, is liberated by the HIV protease and distributed within the assembled viral core (~20%) and in the intravirion fluid (~80%). Upon fusion, the majority of the GFP signal is lost, but the remainder is trapped within the intact core. The progression of reverse transcription causes a change in structure that allows the fluid phase marker GFP to diffuse away from the core. The time between fusion (first GFP signal loss) and second loss defined the time to initiation of uncoating. This method was validated by Infectious Virus Tracking (IVT), which monitors through time-lapse a culture infected with the fluid phase labeled HIV particles at hyper-low MOI where there is less than one virion per cell (8). By imaging many cells under the described conditions, infected cells can be detected, and the behavior of the single virion in the cell that ultimately got infected revealed that only viral particles that initiated uncoating ~30 min after fusion went on to productively infect the cells (8, 48). Here, we employed the Capsid Integrity Assay in CHOpgsA-745 cells treated with HU or COH29 using thoroughly characterized (Fig. S5) dual-labeled (Fluid phase HIV-iGFP and OptiGag-mRuby3-IN) VSV-G pseudotyped HIV-1 and determined if there was modulation of the dynamics of the initiation HIV-1 core uncoating. In the control condition, we performed live-cell imaging without any RNR inhibitor treatment of the cells.

**Fig 5:**
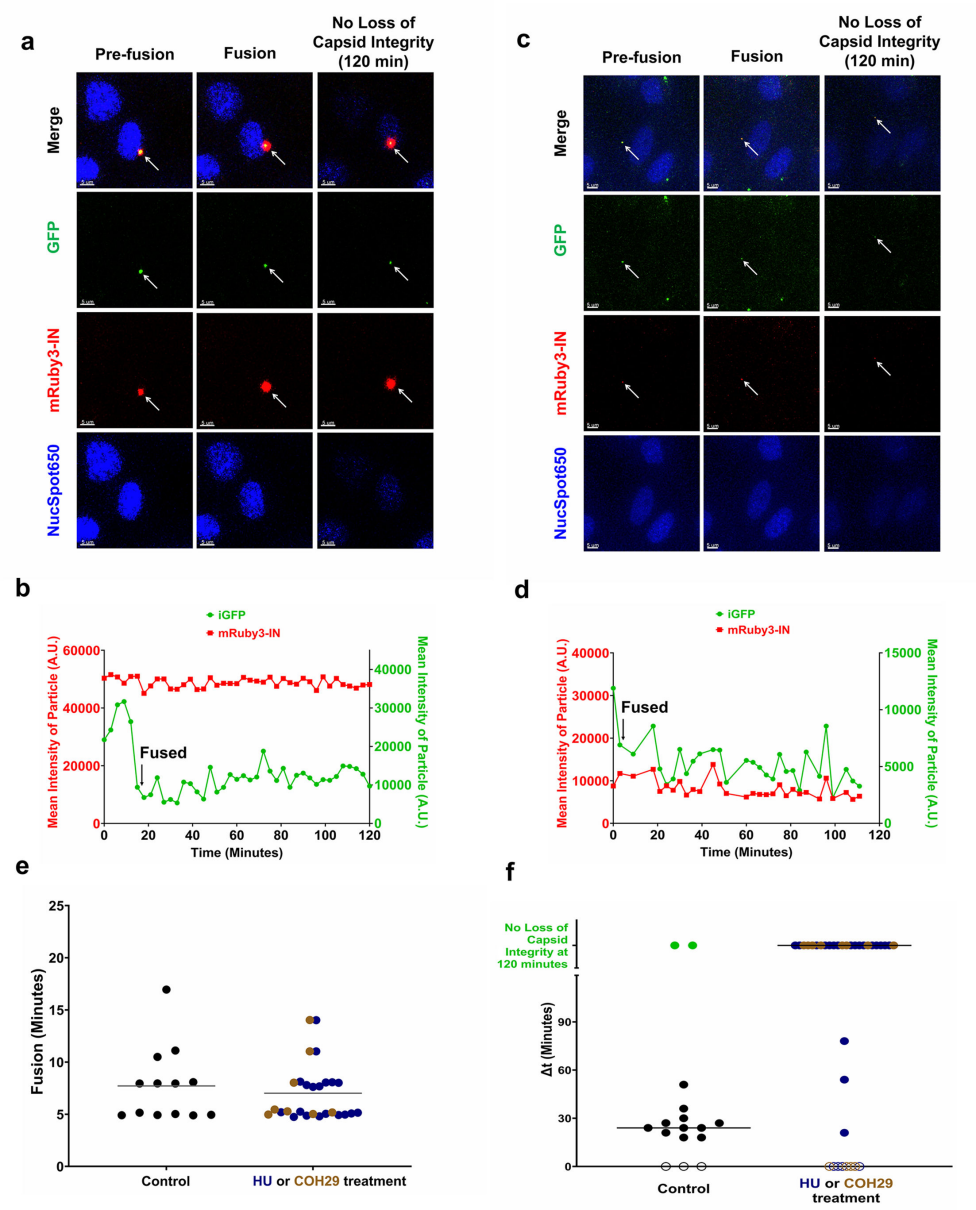
CHOpgsA-745 cells treated with RNR inhibitors delay HIV-1 core initiation of uncoating. (**a, b, c, d**) Representative images from the capsid integrity assay in CHOpgsA-745 cells treated with 1,000 µM HU (a, Movie S2) or 25 µM COH29 (c, Movie S3). Quantification of intensity over time corresponding to the images shown in panels a and c, respectively. The sharp decline in GFP signal denotes viral fusion events, while a complete loss of GFP signal associated with mRuby3-IN indicates the initiation of HIV-1 core uncoating. Data represent one of at least 30 independent events. Arrow in panels a and c indicates the fluorescently labeled viral particle of interest across different channels. Scale bar: 5 µm. (**e, f**) Comparative analysis of the time of fusion and the time interval (Δ*t*) between fusion and capsid integrity loss in cells treated with RNR inhibitor vs untreated controls. Particles that lost GFP signal entirely at fusion are assigned a value of “0” and represented by open circles. Gray horizontal bars indicate median values.

Through the analysis of cells under all conditions, we quantified approximately 50 fusion events, revealing three behaviors of capsid integrity loss: (i) particles that lose all GFP at fusion suggesting they do not have intact cores and are defective (62), (ii) particles that lose the core integrity approximately 30 min after fusion, or (iii) particles never lose the intracore fluid phase GFP within the 120 min of this time-lapse experiment (Fig. 5f). The time to the first drop in fluid phase took place ~10 min on average after starting the time-lapse imaging (Fig. 5e). The first drop in GFP signal detects the fusion of the viral particle as previously described (8), while retaining a subset of GFP inside the intact core until the integrity is lost and the retained GFP diffuses away. In the control condition where CHOpgsA-745 cells did not receive any RNR inhibitor treatment, the results were very similar to our previous report (8). Most of the particles had a phenotype with the loss of GFP signal trapped inside the intact core within approximately 30 min post-fusion (Fig. 5f; Fig. S6a and b; Movie S1). We also observed a small subset of HIV-1 particles losing their entire GFP signal immediately after fusion, as well as a very small portion of the particles not losing GFP signal post-fusion during the experiment, as we previously reported (Fig. 5f) (8). In contrast, CHOpgsA-745 cells treated with HU (Fig. 5a and b; Movie S2) or COH29 (Fig. 5c and d; Movie S3) had a very different outcome where almost all the particles retained the fluid phase marker for the 120 min duration of the experiment. The RNR inhibitors could delay the initiation of uncoating as documented in both assays. This observation is consistent with the delayed reverse transcription kinetics observed after HU and COH29 treatment.

**Fig 6:**
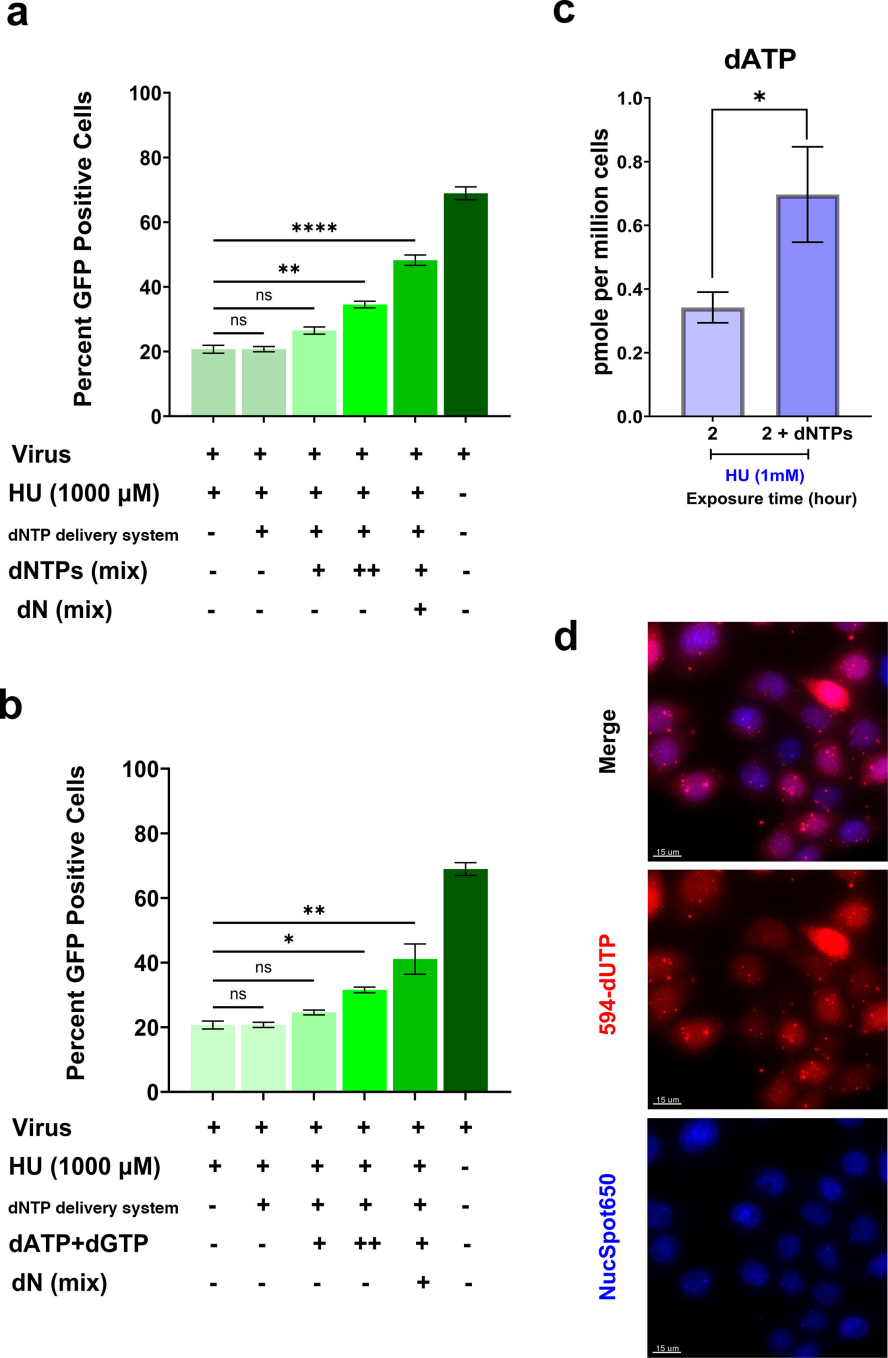
Restoration of HIV-1 infectivity in HU-treated CHOpgsA-745 cells with external delivery of dNTPs. (**a, b**) In the cell-based infectivity assay, external unlabeled dNTPs were delivered using the BioTracker NTP-Transporter under RNR inhibition (HU, 1,000 µM) in CHOpgsA-745 cells. Single and double deliveries were performed (a: 7.5 µM dATP & dGTP, 4 µM dCTP, 1 µM dTTP; b: 10 µM dATP & dGTP), and HIV-1 infectivity restoration was assessed relative to continuous HU treatment. Additional conditions included BioTracker mock delivery, nucleoside (500 µM each) supplementation following a single dNTP delivery under HU pressure, and virus-only controls. Statistical significance was assessed using one-way ANOVA with Dunnett’s post-hoc correction, comparing each treatment group to the continuous HU treatment control. Significance levels: ****P  <  0.0001, **P  =  0.0015, *P  =  0.0138, ns  =  non-significant. For dN and external dNTP delivery: “+” indicates a single delivery, while “++” indicates two deliveries. In all other cases, “+” denotes presence, and “−” denotes absence. Data are representative of three independent biological replicates (data from repeats are shown in Fig. S7); error bars denote SD among technical repeats. (**c**) External dATP (10 µM) was delivered via the BioTracker NTP-Transporter in CHOpgsA-745 cells treated with HU (1,000 µM, 2 h), and dATP level was compared to HU-only treatment using an unpaired *t*-test (*P = 0.0333). Error bars represent SE from three independent biological replicates. (**d**) Fluorescence microscopy confirmed BioTracker-mediated delivery of labeled dUTP (10 µM) in CHOpgsA-745 cells, demonstrating uptake across the cell population. Scale bar: 15 µm.

To assess whether the observed delay in the initiation of uncoating is due to any off-target effects of the inhibitor, we attempted to restore the HIV-1 infectivity by externally delivering an unlabeled dNTPs mixture using BioTracker NTP-Transporter. This was performed in the presence of a relatively high concentration of HU (1,000 µM) in CHOpgsA-745 cells challenged with a high viral load, which resulted in approximately 20% baseline infectivity. HIV-1 infectivity was significantly restored with two consecutive dNTP deliveries and nucleoside feeding after a single dNTP delivery compared to the continuous RNR inhibitor (HU) treatment control. However, HIV-1 infectivity was not significantly restored with a single dNTP delivery or the dNTP transporter alone (Fig. 6a). HU treatment has been shown to predominantly inhibit the *de novo* synthesis of dATP and dGTP in mammalian cells (57, 63); therefore, we repeated the experiment with the delivery of the exogenous dATP and dGTP. As shown in Fig. 6b, two consecutive deliveries of dATP and dGTP significantly restored infectivity compared to the continuous RNR inhibitor (HU) treatment control. To further confirm the restoration of HIV-1 infectivity with external delivery of dNTPs, we delivered 10 µM of dATP using equimolar BioTracker NTP-Transporter in CHOpgsA-745 cells treated with HU (1,000  µM) for 2 h and measured the cellular dATPs using HIV-1 RT-based dNTP assay and found a significant twofold increase in intracellular dATPs compared to HU-treated cells (Fig. 6c), confirming the successful external dNTP uptake and supporting the HIV-1 infectivity restoration data. We also optimized the dNTP delivery system in live-cell imaging set-up by delivering AlexaFluor 594-5-dUTP using equimolar transporter molecules in CHOpgsA-745 cells and observed that almost all the cells received labeled dNTP, with cell-to-cell variability in terms of labeled dNTP uptake (Fig. 6d).

**Fig 7:**
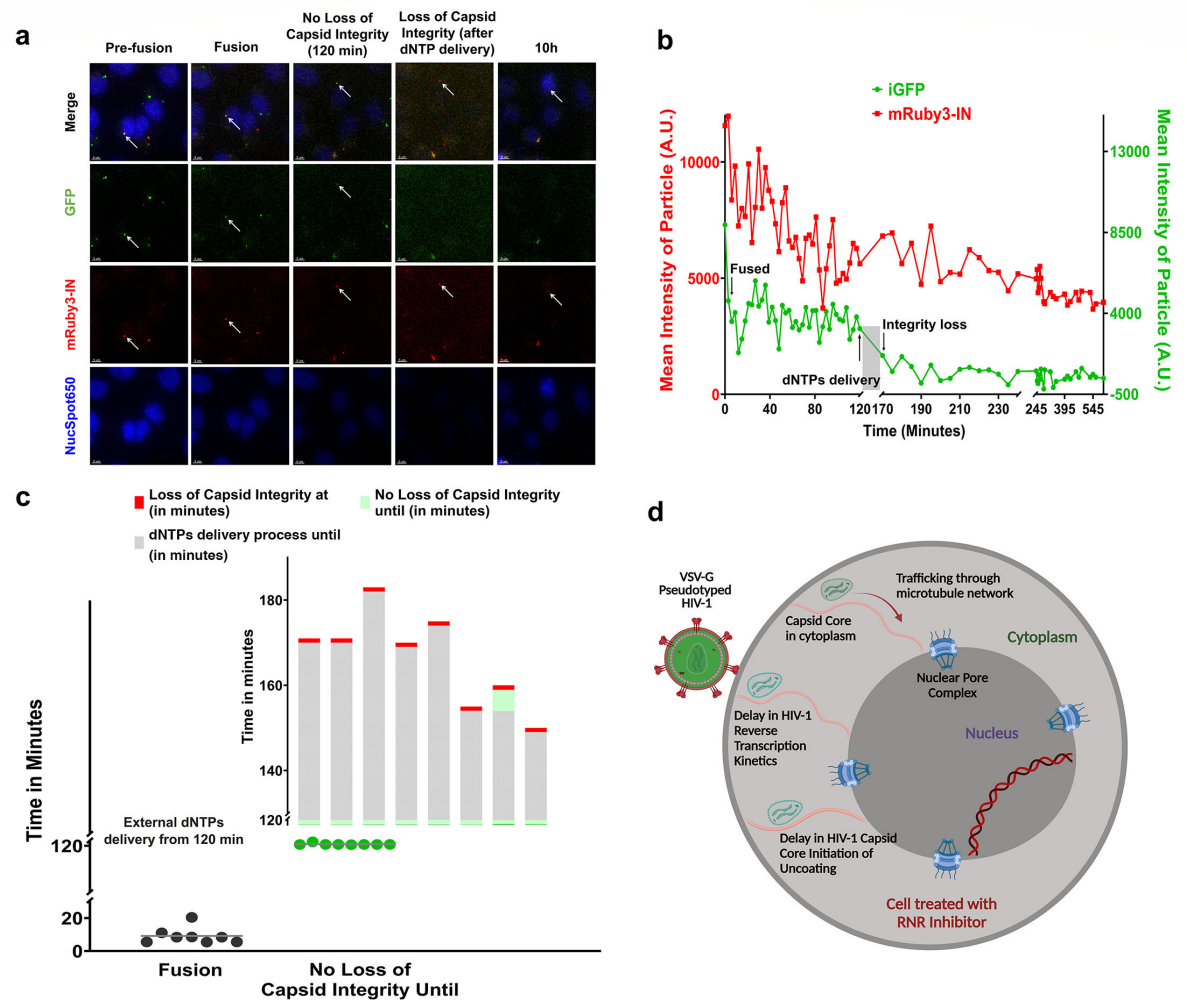
External delivery of dNTPs in HU-treated CHOpgsA-745 cells reinstates HIV-1 core initiation of uncoating. (**a, b**) Representative images from the capsid integrity assay in CHOpgsA-745 cells treated with HU (1,000 µM, 2 h) followed by immediate delivery of unlabeled dNTPs (2.5 µM each) using BioTracker NTP-Transporter while maintaining HU pressure. Quantification of intensity over time corresponds to the images in (**a**). A sharp decline in GFP signal indicates viral fusion, while complete loss of GFP associated with mRuby3-IN (**b**) marks HIV-1 core initiation of uncoating (Movie S4). Data represent one of at least eight independent events. Arrow in (a) indicates the fluorescently labeled viral particle of interest across different channels. Scale bar: 5 µm. (**c**) dNTP delivery was performed over 15-40 min, with total processing, including washing steps, taking 30–60 min. Time-lapse imaging was temporarily paused during delivery to reduce background noise from the dNTP buffer. Eight events were tracked, showing rapid GFP loss within 10 min post-fusion, with residual GFP retaining intact cores for at least 120 min under HU treatment. Capsid integrity loss, indicated by post-fusion GFP disappearance, occurred following dNTP delivery despite continued RNR inhibition. (**d**) Model of HIV-1 early infection dynamics in cells with reduced dNTP Pool due to RNR inhibition. In cells, HIV-1 undergoes capsid core transport and trafficking toward the nucleus after fusion. However, in cells with a reduced dNTP pool achieved by blocking the *de novo* pathway of dNTPs biosynthesis by RNR inhibition, we observed delays in HIV-1 reverse transcription kinetics and the initiation of capsid core uncoating. Designed in https://BioRender.com.

After optimizing the external dNTP delivery pipeline, we examined whether external dNTP delivery could reinstate HIV-1 core initiation of uncoating during live cell imaging assay. Following 120 min of time-lapse imaging with dual-labeled VSV-G pseudotyped HIV-1, as previously described, we delivered unlabeled dNTPs in HU-treated CHOpgsA-745 cells while maintaining HU pressure. The incubation period for dNTP delivery ranged from 15 to 40 min, with the entire process, including upstream and downstream washing and adding steps, taking approximately 30–60 min. Due to the periodic washing steps and increased background noise from the dNTP delivery buffer, we temporarily paused time-lapse imaging during the delivery process and resumed it afterward. We tracked single HIV-1 particles post-fusion for their GFP loss dynamics before and after external dNTP delivery. In HU-treated CHOpgsA-745 cells, HIV-1 particles that retained subsets of GFP within intact cores during the first 120 min of live imaging resumed core integrity loss after the external delivery of unlabeled dNTPs (indicated by the loss of post-fusion GFP trapped inside the intact core) (Fig. 7a and b; Movie S4). This core integrity loss occurred across varied dNTP delivery periods (30–60 min) despite continued HU treatment (Fig. 7c). Our findings demonstrate that limited intracellular dNTP availability slows HIV-1 reverse transcription and delays the initiation of core uncoating. The restoration of these processes upon exogenous dNTP delivery underscores the critical role of adequate dNTP pools in facilitating efficient HIV-1 replication.

## DISCUSSION

The progression of events that initiate when an HIV-1 core containing two copies of the RNA genome is introduced into the cytoplasm after fusion and culminates in the formation of a double-stranded DNA genome that is integrated into the host cell genome is complicated, flexible, and adaptable to very different intracellular environments. The successful completion of reverse transcription required to generate the DNA provirus relies on the availability of deoxynucleotide triphosphates (dNTPs) supplied by the host cell (1, 9, 11). This complex and highly coordinated process taking place in the core must also navigate a minefield of restriction factors and host cell regulation of intracellular metabolites and physiology that make the cellular environment hostile to pathogens (1, 49). Such essential metabolites include dNTPs, which are required primarily during DNA replication. Many HIV-1 target cells *in vivo*, such as macrophages, dendritic cells, and resting CD4+ T cells contain low levels of dNTPs (11–14, 16). This is reflected in an increase in the time required for the completion of reverse transcription in such cell types relative to other cells with abundant dNTP pools (11–14, 16).

To gain insights into how the inhibition of the *de novo* pathway of dNTP biosynthesis impacts the various steps of the early HIV-1 lifecycle, we targeted key players in dNTP metabolism with inhibitors. Multiple drugs inhibit RNR and disrupt the *de novo* pathway (33, 35, 55, 56). RNR inhibitors decrease the rate of the completion of reverse transcription as revealed by the loss of sensitivity to NVP. In both CHOpgsaA-745 and TZMbl cells treated with HU or COH29, we observed a compromise in HIV-1 reverse transcription kinetics (Fig. 3b and c; Fig. S2c and d). We interpret the observed compromise in HIV-1 reverse transcription kinetics as a consequence of the reduction in the host cell dNTP pool size (Fig. 2), which hampers the processivity of HIV-1 RT (64). Previous studies have reported that HU treatment reduces the cellular dNTP pool to lower levels (34, 57). Similarly, a more pronounced reduction of the cellular dNTP pool after treatment with COH29 has been documented (33, 35). These inhibitors also reveal the presence of backup pathways, referred to as salvage pathways, to supply dNTP into the cellular dNTP pool by converting membrane-soluble nucleosides into nucleotides (11, 20), thus allowing the completion of reverse transcription at delayed time points (Fig. 2a; Fig. S2b).

One clear outcome of these studies is that both the homeostasis of intracellular dNTPs and the progression of the viral core to generate an integrated DNA genome are dynamic and adaptable. The inhibition of the *de novo* pathway with different RNR inhibitors rapidly generated a state where sensitivity to an inhibitor of reverse transcription was extended by hours, consistent with the notion that the depletion of the dNTP pool slows the completion of reverse transcription (65). This response to RNR inhibitors revealed the known rapid depletion/reduction of dNTP pools (Fig. 2). Even though the inhibitors were added to the cultures after synchronized infection with the virus, the impact on reverse transcription was immediately manifested. To gain further insights into the dynamics of this system, we evaluated the impact of the dNTP hydrolase, SAMHD1. Degradation of SAMHD1 with Vpx only partially restored the rate of completion of reverse transcription (Fig. S4e) consistent with other players in dNTP turnover, such as 5′-nucleotidases (5′-NT) (20). The impact of SAMHD1 depletion partially reversing the timing of sensitivity to reverse transcription inhibition is consistent with RNR inhibition rapidly leading to a decrease in dNTP pools. Evaluation of the relationship between RNR inhibition by HU treatment and changes in SAMHD1 expression and intracellular localization revealed a dynamic system. SAMHD1 can be distributed in the cytoplasm, nucleus, and nuclear structures in a phosphorylation-dependent manner (17). We observed a change in the SAMHD1 nuclear localization after treatment with RNR inhibitor (HU) and aphidicolin, both of which cause cell-cycle arrest in the entry to the S phase (66). Both treatments induce the DNA damage response (67) punctuate diffraction-limited nuclear structures (68) identified as a significant increase in the intensity and number of SAMHD1 nuclear spots (Fig. S4a through c). Previous report indicates this new distribution may be the localization of SAMHD1 to replication forks (69). SAMHD1 protein levels are minimal during the S-phase and maximal during quiescence. SAMHD1 is also phosphorylated over the cell cycle. The phosphorylation at Thr592 by cyclin-dependent kinases inhibits nucleotide hydrolysis activity and is maximal during S phase (70). This phosphorylation also plays a role in SAMHD1 localization (71). Our studies of SAMHD1 expression levels and localization revealed a dynamic inter-relationship between dNTP pools and dNTP catabolism to function optimally over the cell cycle.

The RNR inhibitors are reversible. A simple washout revives the host cell dNTP pool, thereby facilitating the restoration of HIV-1 infectivity (Fig. 1b, e, 3b and c; Fig. S2c and d). The ability to recover infectivity almost to control levels also provides evidence that the timing of the initiation of HIV-1 uncoating and the delay of reverse transcription does not lead to an abortive status. Instead, infectious-capable virions are still able to ultimately complete reverse transcription and integrate under these depletion/restoration conditions. The reverse transcribing core is clearly adaptable to variable dNTP pools as would be expected to be encountered in primary target cells. The sensitivity of the capsid integrity assay to RNaseH inhibitors, and the rapid loss of core integrity observed after washout (8) suggest that the initiation of uncoating happens very early in the process of reverse transcription, soon after the first jump which primes reverse transcription after strong-stop anneals with complementary sequences in the 3′ genomic RNA sequence (8, 72).

Our current interpretation of the results presented here revealed the different RNR inhibitors only decreased dNTP pools without any off-target drug effects or toxicity (Fig. 2; Fig. S1a). Consistent with RNR inhibitors slowing the completion of HIV-1 reverse transcription by causing a reduced dNTP pool, it was possible to reverse the RNR inhibitor phenotype with treatments that increase intracellular dNTP pools. For example, in the dNTP rescue experiment conducted in CHOpgsA-745 cells, we observed a partial but significant rescue of HIV-1 infection following the introduction of nucleosides into the culture media (Fig. 1c, f and 2a). It was also possible to increase the HIV-1 infectivity of cells treated with RNR inhibitors by external delivery of dNTPs (Fig. 6a through c). Likewise, intact HIV-1 cores containing the GFP fluid phase marker can be stimulated to lose integrity and leak GFP after liposomal dNTP delivery when allowing reverse transcription to proceed and signal the core to initiate the process of uncoating (Fig. 7a and b). Together, these observations suggest that the different RNR inhibitors are specifically acting to reduce intracellular dNTP pools because the slowing of the process of reverse transcription by decreasing dNTPs is rapidly reversible, partially restored with nucleosides (salvage pathway), and complemented to a significant extent with delivered dNTPs. These results suggest that while salvage pathways play a crucial role in sustaining cellular dNTP levels, they may have limitations in fully compensating for the loss of *de novo* dNTP biosynthesis pathway through RNR inhibition (73)

Further insights into the dynamic nature of this system maintaining and regulating dNTP homeostasis were revealed by the differential ability of the RNR inhibitors to completely block HIV-1 infectivity through inhibition of *de novo* pathway (Fig. S1c). These findings underscore the differential effectiveness of RNR inhibitors in modulating HIV-1 infection and reveal known variations in their regulation and mechanisms of action (33, 35, 55, 56). RNR is composed of a large subunit (α/RRM1) that is constitutively expressed and a variable and tightly regulated small subunit (β-subunit). There are two forms of RNR small subunits, RRM2 and RRM2B (also known as p53R2) which are differentially controlled. RRM2 is regulated over the cell cycle, with peak expression during the S phase to provide necessary dNTPs. RRM2B is primarily induced by p53 activation under stress conditions providing smaller amounts of dNTPs for mitochondrial DNA replication and DNA repair during the p53 response to DNA damage (29). RRM2B is also able to function to produce low levels of dNTPs in hypoxic conditions (31). The presence of background infection in HU-treated cells with high titer virus challenge (Fig. 6a and b; Fig. S1c), despite using higher inhibitor concentrations, could be attributed to the more pronounced inhibition of RRM2, but not RRM2B, by HU (36, 37). In contrast, the broader inhibitory spectrum of 3-AP and COH29, which target both the RRM2 and RRM2B small subunits of mammalian RNR, can completely block HIV infection at high concentrations as shown in Fig. S1c (33, 35–37).

In the cell-based infection assay, external delivery of one application of dNTPs into CHOpgsA-745 cells (HU-treated) under different conditions did not significantly restore HIV-1 infectivity. In contrast, the external delivery of two applications of dNTPs, along with the addition of nucleosides to boost salvage pathways, significantly restored HIV-1 infectivity (Fig. 6a and b). It has been previously reported that HU treatment of CHOpgsA-745 cells led to an approximately 90% reduction in the whole cell pool of dATP and dGTP (57). In our experimental condition with HU-treated CHOpgsA-745 cells, followed by delivering exogenous dATP (10 µM) & dGTP (10 µM) as well as a 4 dNTPs mixture (7.5 µM each of dATP & dGTP, 4 µM of dCTP, and 1 µM of dTTP) significantly restored HIV-1 infection. Compared to the restoration of HIV-1 infectivity achieved by washing out the RNR inhibitor (Fig. 1b and e) and boosting the salvage pathways with exogenous nucleoside application feeding (Fig. 1c and f) in RNR inhibitor-treated CHOpgsA-745 cells, the effect of external dNTP delivery of HIV-1 infectivity, particularly when administered once, was not significant (Fig. 6a and b). In pilot studies, we observed that a single delivery of fluorescent dNTPs revealed that the BioTracker NTP-Transporter Molecule-based dNTP delivery system was not delivering dNTPs uniformly across the cell population (Fig. 6d). Additionally, the high rate of dNTP turnover would rapidly degrade any exogenously delivered dNTPs. But when we tried to increase the concentration of the delivered dNTPs or increase the amount of the BioTracker NTP-Transporter, we observed toxicity which led us to try a second dNTP delivery. A second delivery of exogenous dNTPs was able to introduce enough dNTPs to allow the completion of reverse transcription and subsequent infection (Fig. 6a and b). Even though the inefficiency of the dNTP delivery system was not able to deliver as much as dNTPs necessary to meet the demand of the dNTP-starved cells to fully restore HIV-1 infection to control levels, the experiment does demonstrate that the cellular environment can be permissive to HIV-1 infection when exogenous dNTPs are provided. This outcome demonstrates the importance of an active *de novo* pathway of dNTP biosynthesis to maintain homeostasis, consistent with recent findings indicating that the ability of Vpx to overcome SAMHD1’s restriction requires the *de novo* pathway in monocyte-derived macrophages (MDMs) to restore susceptibility to HIV-1 infection (9). Hence, further research is warranted to elucidate the complex interplay between HIV-1 infection and the host cell dNTP pools provided by *de novo* and salvage pathways.

The rescue experiments with dNTPs and nucleosides and the resulting partial recovery (Fig. 1c, f, 6a and b) suggest that the salvage pathways can independently counteract the dNTP depletion induced by RNR inhibition and restore cellular dNTP pools to levels that can effectively support HIV-1 replication, but with slower kinetics. Despite our efforts to enhance salvage pathways in CHOpgsA-745 cells by supplying nucleoside precursors, we noted that the depletion of dNTPs induced by RNR inhibition could not be entirely reversed because the kinetics of the completion of reverse transcription did not return to control cell levels. This is likely because the cellular dNTP pool did not reach sufficient levels without an active *de novo* pathway (Fig. 1c, f and 2a). The system is further modulated by the dNTP hydrolase SAMHD1, which is highly regulated at the level of expression with its enzymatic function regulated by phosphorylation. However, SAMHD1 is only partially responsible for the observed dNTP turnover because targeting SAMHD1 for proteasomal degradation by the addition of Vpx, only increased the kinetics of the completion of reverse transcription as shown in Fig. S4e. Therefore, SAMHD1 can fine-tune dNTP levels to maintain homeostasis under variable conditions.

The change in the time to completion of reverse transcription appears to represent a general slowing of the rate of the entire process because our observed delay in the earliest steps of the HIV-1 early life cycle is reflected in a delay in the time needed for the maturation and reorganization of the core to become resistant to restriction by TRIM-CypA (Fig. 4c). Likewise, the time in the initiation of uncoating, as detected microscopically as the loss of fluid phase GFP contained within the core, is also delayed (Fig. 5a through d). These delays in the reorganization and remodeling of the HIV-1 core are happening very soon after the entry of the core into the cytoplasm. The diminished dNTP pools are low enough to delay the completion of HIV-1 reverse transcription by hours, but the population becomes resistant to the inhibition after extended periods of culture (Fig. 3b and c; Fig. S2c and d). This kinetic restriction of reverse transcription in different cell types is observed *in vivo* when a macaque is infected with SIV/SHIV expressing a Vpx with mutations that specifically prevent the ability of Vpx to target a ubiquitin ligase complex to SAMHD1 for ubiquitination and subsequent proteasomal degradation (74). The phenotype of this virus is a lack of infection of tissue-resident CD4 T cells. Myeloid cell populations are still infected. Therefore, the restriction observed in tissue culture is kinetic in the myeloid cells which are eventually infected in contrast to the resting T cells which are not infected. This change in the kinetics of reverse transcription and uncoating demonstrates the flexibility and adaptability of the virus to replicate in hostile cellular intracellular environments that attempt to starve the virus of essential metabolites such as dNTPs. Future studies will seek to determine how RNR inhibition will alter the kinetics of other steps such as nuclear localization and integration. These studies may reveal other ways that the virus can adapt to this dNTP-depleted environment to complete the essential process of reverse transcription while avoiding restriction factors.

This phenomenon suggests that HIV-1 RT has evolved to adapt to suboptimal cellular dNTP pools over time and can complete the process of reverse transcription, albeit with slower kinetics, in this hostile, antiviral environment (Fig. 3b and c; Fig. S2b through d). Generally speaking, all of the primary targets for HIV-1 *in vivo* carry low levels of dNTPs including, tissue-resident CD4 cells, TH17 cells, dendritic cells, macrophages, Langerhans cells, and mast cells are primarily in a quiescent or resting state. This observation is consistent with a previous body of research describing lentiviral RTs, including HIV-1, as exhibiting exceptional efficiency even at low dNTP levels compared to other retroviruses (75–77). It is known that HIV-1 infection of macrophages is slowed by naturally reduced endogenous dNTP pools relative to higher dNTP pools in activated CD4 T cells and transformed tissue culture models (78). Based on the results presented in this manuscript, RNR treatment turns the tissue culture cell lines into cells with a macrophage-like dNTP pools (Fig. 7d). These are major differences to studies of the uncoating of HIV-1 in macrophages, where results have been interpreted to reveal that intact cores can be observed in the nucleus. However, the difference in the dNTP pools in macrophages may make those cells highly sensitive to dNTP levels, causing intact cores to enter the nucleus prior to uncoating. The addition of RNR inhibitors is simple and can be utilized in diverse laboratories studying the early steps of the HIV-1 lifecycle to gain additional insights to explain the disparate results emerging from different labs studying HIV-1. It is also notable that in the early days of the HIV pandemic, HU was utilized as a treatment, often in combination with compounds that inhibited HIV-1 enzymes protease or RT (38, 39). The impact of HU on cellular dNTP levels could increase the potency of other compounds. Future strategies for HIV treatment may be able to utilize RNR inhibition as part of a targeted intervention for HIV-1 eradication.

## SUPPLEMENTAL MATERIAL

Supplemental material mbio.01047-25-s0001.pdf In-depth methodological description, and supplemental figures, and legends for supplemental movies.

Movie S1 mbio.01047-25-s0002.mp4 Live-cell imaging of HIV-1 core initiation of uncoating in RNR inhibitor untreated CHOpgsA-745 cells reveals GFP loss post-fusion.

Movie S2 mbio.01047-25-s0003.mp4 HU treatment delays HIV-1 core initiation of uncoating in CHOpgsA-745 cells, with prolonged post-fusion GFP retention.

Movie S3 mbio.01047-25-s0004.mp4 COH29 treatment delays HIV-1 core initiation of uncoating in CHOpgsA-745 cells, with prolonged post-fusion GFP retention.

Movie S4 mbio.01047-25-s0005.mp4 Delayed HIV-1 core initiation of uncoating in HU-treated cells is reversed by external dNTP delivery.

ASM does not own the copyrights to Supplemental Material that may be linked to, or accessed through, an article. The authors have granted ASM a non-exclusive, world-wide license to publish the Supplemental Material files. Please contact the corresponding author directly for reuse.
