# Supplementary material for: Blockade of de novo dNTP biosynthesis pathway delays HIV-1 early life cycle kinetics and dynamics
Source: mBio. 2025 Jun 30;16(8):e01047-25. doi: 10.1128/mbio.01047-25 (PMC12345267; doi:10.1128/mbio.01047-25)
Supplement: Supplemental material — In-depth methodological description, and supplemental figures, and legends for supplemental movies. [file mbio.01047-25-s0001.pdf]

Supplemental Material

In-depth methodological descriptions

Methanol-based Metabolite extraction for dNTP quantification

CHOpgsA-745 cells (final density 1 to 3 million cells) were washed twice with PBS or ice-cold saline and lysed directly on the plate by adding ice-cold methanol. Cells were thoroughly scraped, and the lysate was transferred to a tube, followed by vigorous vortexing. Cells were completely lysed either by repeated freeze-thaw cycle or heating at 95°C followed by immediate chilling on ice. After centrifugation at 14,000 RPM, the methanol supernatant was collected and dried under vacuum. Dried extracts were stored at −80°C until dNTP quantification by HIV-1 RT-based dNTP assay and LC-MS/MS based targeted metabolomics.

LC-MS/MS-based Quantification of Cellular dNTPs

Cellular dNTP levels were quantified using liquid chromatography–tandem mass spectrometry (LC-MS/MS) performed at the Metabolomics Core Facility, Northwestern University. After reconstitution of samples in 60% acetonitrile, analyses were conducted on a Thermo TSQ triple quadrupole mass spectrometer equipped with an electrospray ionization (ESI) source, coupled to a Thermo Vanquish UHPLC system with a binary pump, degasser, and autosampler. Chromatographic separation was achieved using an XBridge C18 column (2.1 mm × 100 mm, 3.5 µm particle size, Waters) operated in isocratic mode with a mobile phase consisting of 95% solvent A (10 mM ammonium formate, 0.1% formic acid in water) and 5% solvent B (acetonitrile), at a flow rate of 0.15 mL/min. The ESI source was operated in positive ion mode with a capillary temperature of 300 °C, sheath gas at 35 arbitrary units, auxiliary gas at 3 arbitrary units, and a spray voltage of 3.5 kV. Selective reaction monitoring (SRM) transitions were used to detect the protonated precursor-to-product ion transitions for dATP (m/z 491 → 136), dGTP (m/z 508 → 152), dCTP (m/z 468 → 112), dTTP (m/z 483 → 81), and dUTP (m/z 469 → 81). Peak integration and quantitative data analysis were performed using Xcalibur 4.1 and TraceFinder 4.1 software (Thermo Fisher Scientific).

Immunofluorescence staining of SAMHD1

To analyze the SAMHD1 status of the cells under study, CHOpgsA-745, TZMbl, and OMK cells were seeded on 12mm round coverslips (No-1.5). Besides, the coverslip was treated with Fibronectin and seeded with THP-1 cells without activation. After that, cells were fixed with PIPES buffer and 3.7% formaldehyde, followed by blocking with 3% BSA. Cells were then permeabilized with 0.5% Triton X-100 treatment. SAMHD1 staining was performed with a 1:500 dilution of anti-SAMHD1 antibody (ab128107), and SAMHD1 expression was detected using an anti-mouse Donkey Rhodamine Red™-X (RRX) AffiniPure Fab Fragment (715-297-003). After DAPI staining, slides were prepared for imaging.

Quantification of SAMHD1 nuclear spot and intensity

Quantitative assessment of the RNR inhibition-induced changes in SAMHD1 expression in CHOpgsA-745 cells was conducted by measuring SAMHD1 nuclear speckle-like signal or spot. To achieve this, the DAPI channel was segmented using the Cellpose (1) plugin annexed into QuPath software version 0.4.3 (2). SAMHD1 nuclear speckle-like signal or spot, SAMHD1 nuclear signal intensity sum, and the mean intensity of the SAMHD1 nuclear signal was quantified using QuPath's sub-cellular detection plugins.

SIV Vpx VLP treatment and Nevirapine addition assay

CHOpgsA-745 cells were treated with HU (250 µM) overnight to induce SAMHD1 nuclear redistribution, simultaneously with Vpx VLP treatment to degrade SAMHD1. After the removal of HU and overnight incubation to restore the cells, an NVP addition assay was performed as described earlier and shown in Figure S4d.

Supplemental Figures

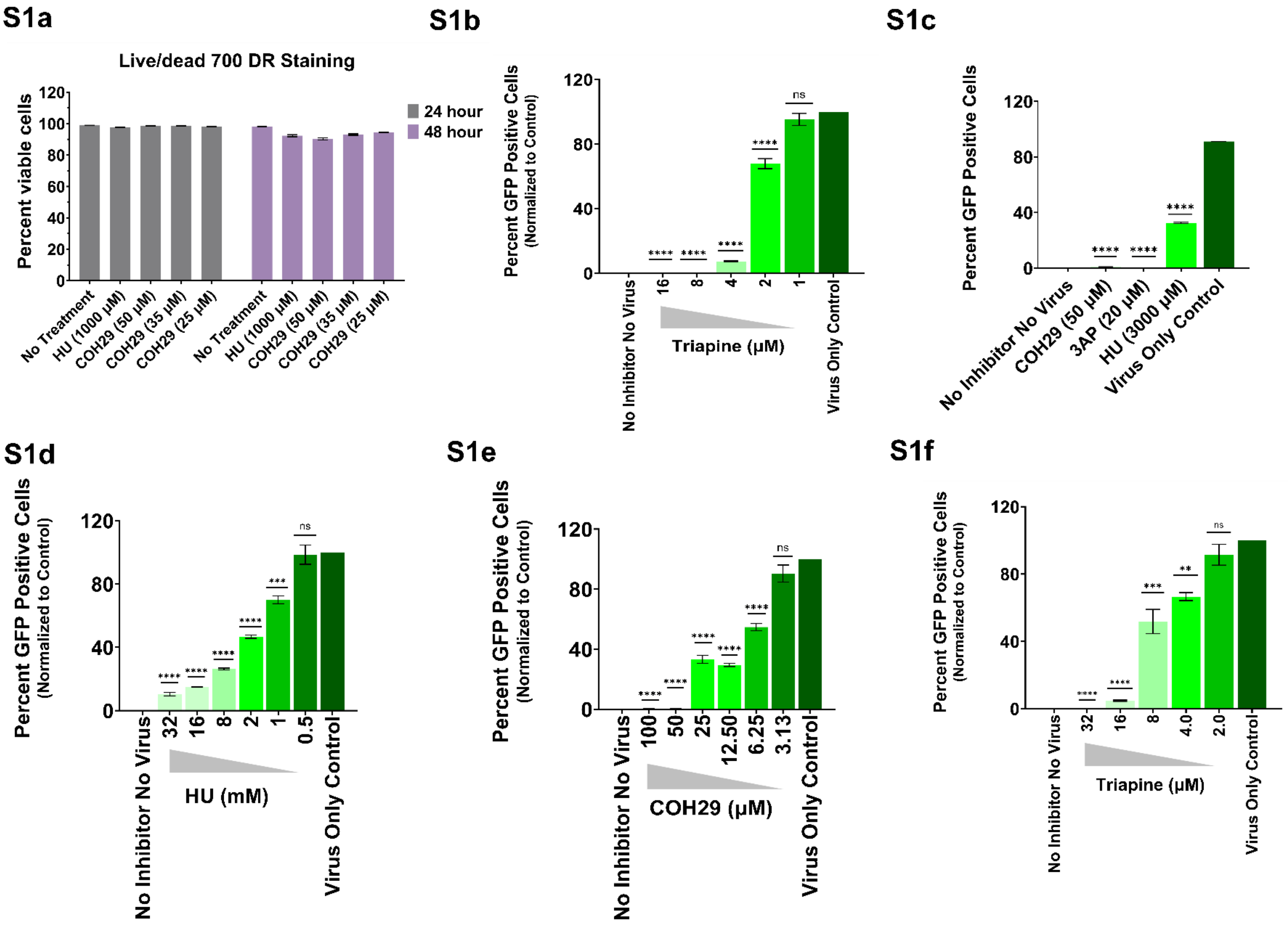

Supplemental Figure S1. Host Cell treated with RNR Inhibitors Reduce HIV-1 Infectivity in different cell lines.

S1a) Cell viability among the population was accessed after 24 hours and 48 hours of treatment with hydroxyurea (HU) or varying concentrations of COH29 compared to untreated controls.

S1b) Immediately post-synchronized infection, CHOpgsA-745 cells were treated with increasing concentrations of Triapine (3-AP). HIV-1 infectivity was assessed and compared to the virus-only control.

S1c) Immediately post-synchronized infection with a high virus challenge, CHOpgsA-745 cells were treated with HU, 3-AP, and COH29. HIV-1 infectivity was assessed and compared to the virus-only control.

S1d, S1e, S1f) Immediately post-synchronized infection, TZMbl cells were treated with increasing concentrations of HU, COH29, and 3-AP. HIV-1 infectivity was assessed and compared to the virus-only control.

Statistical significance was determined using one-way ANOVA with Dunnett's post-hoc correction to compare each treatment group to the virus-only control. In every case, the bar plots shown (except S1c) are the percentage of GFP positive cells at each condition/concentration of inhibitor normalized by setting the percentage of GFP positive cells at virus only control as 100%. Error bars represent the standard error (SE) from three independent biological replicates. Significance levels are indicated as follows: **** $p < 0.0001$, *** $p = 0.0003$, ** $p = 0.0038$, ns = non-significant.

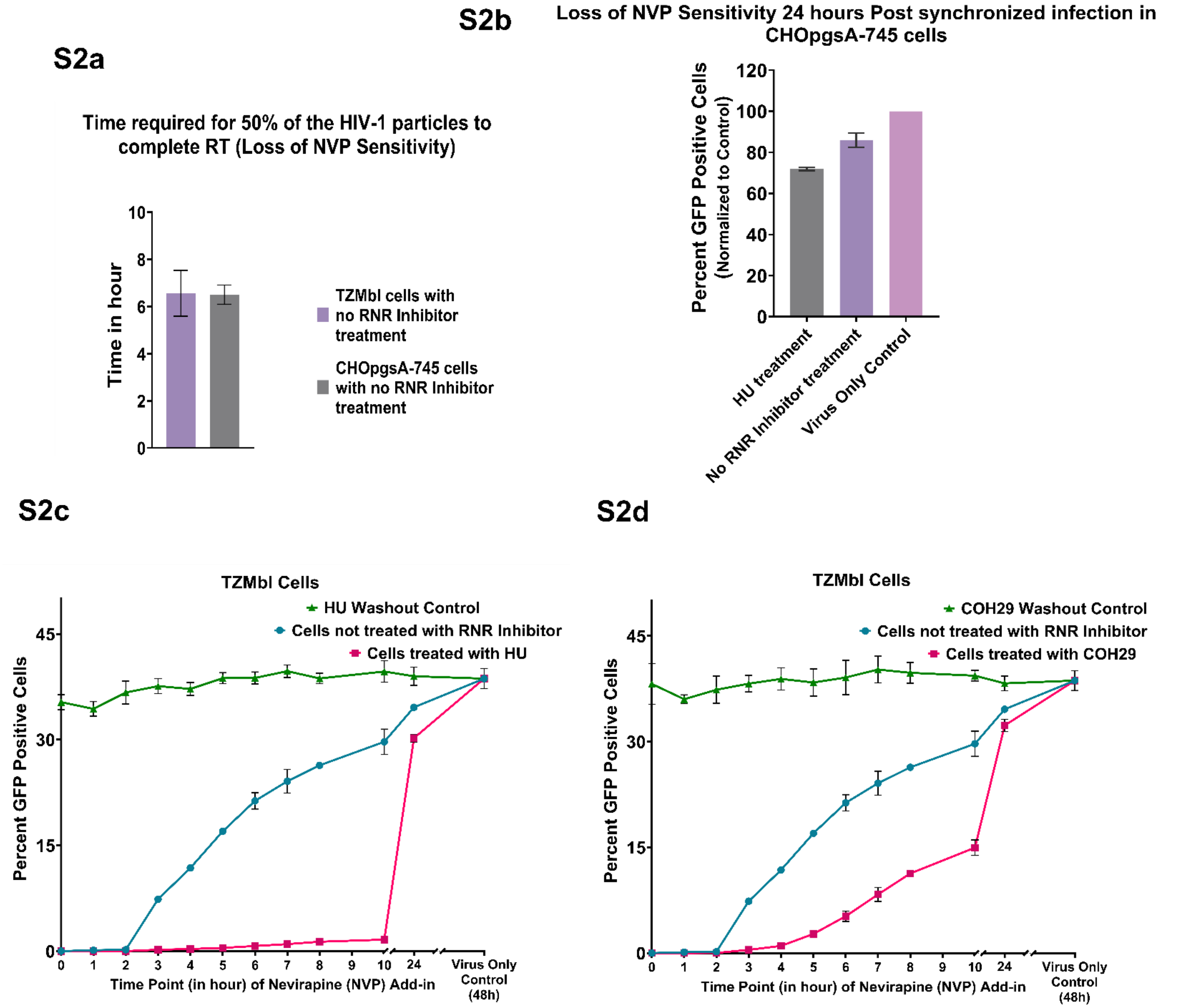

Supplemental Figure S2. Impact of RNR Inhibitor on HIV-1 Infectivity and Reverse Transcription Kinetics in Cell line model

S2a) The curve averaged the time (in hour) required for 50% of the HIV-1 particle to complete reverse transcription (as indicated by the loss of sensitivity to NVP) in CHOpgsA-745 and TZMbl cells that received no RNR inhibitor treatment. At least three independent biological repeats were taken into consideration per condition.

S2b) In CHOpgsA-745 cells, HIV-1 reverse transcription completion (as indicated by the loss of sensitivity to the timed addition of NVP) after 24 h of RNR inhibitor treatment and compared to HIV-1 reverse transcription completion under cellular conditions with no inhibitor treatment. Here the error bar denotes standard error (SE) of three independent biological repeats.

S2c, S2d) Post-synchronized infection, TZMbl cells treated with HU (1000 µM) or COH29 (35.0 µM) for the duration as indicated in (Figure 3a) followed by washing out and immediately replacing with NVP (10 µM) supplemented media. The sensitivity to NVP treatment (HIV-1 Reverse Transcription Kinetics) was accessed and compared to cells that received no RNR inhibitor treatment. Reversibility of RNR inhibition at each-time point was measured by washing out the inhibitor. Data are representative of three independent biological experiments. Error bars indicate the standard deviation (SD) of three technical replicates within this representative experiment.

S3a

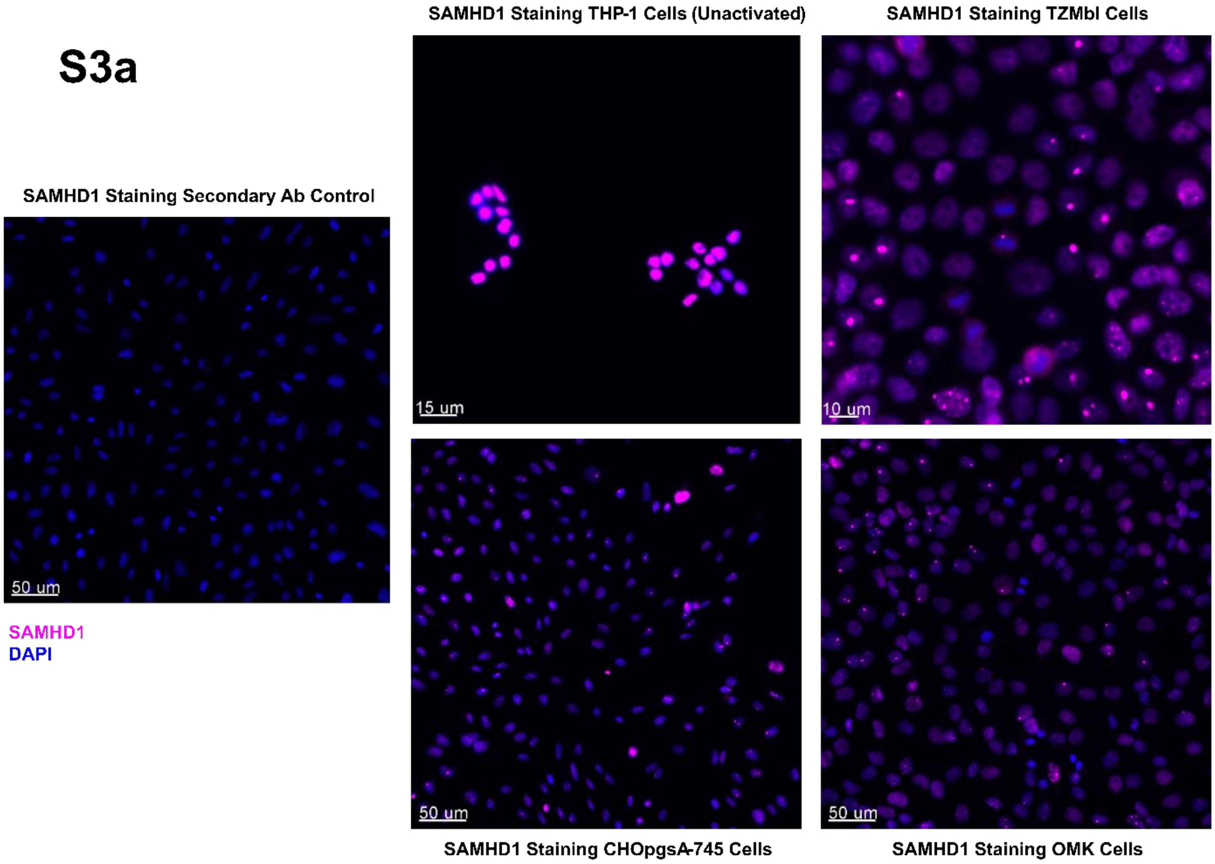
SAMHD1 Staining THP-1 Cells (Unactivated)
SAMHD1 Staining TZMbl Cells
SAMHD1 Staining Secondary Ab Control
15 um
10 um
50 um
50 um
50 um
SAMHD1 Staining CHOpgsA-745 Cells
SAMHD1 Staining OMK Cells
SAMHD1
DAPI

S3b

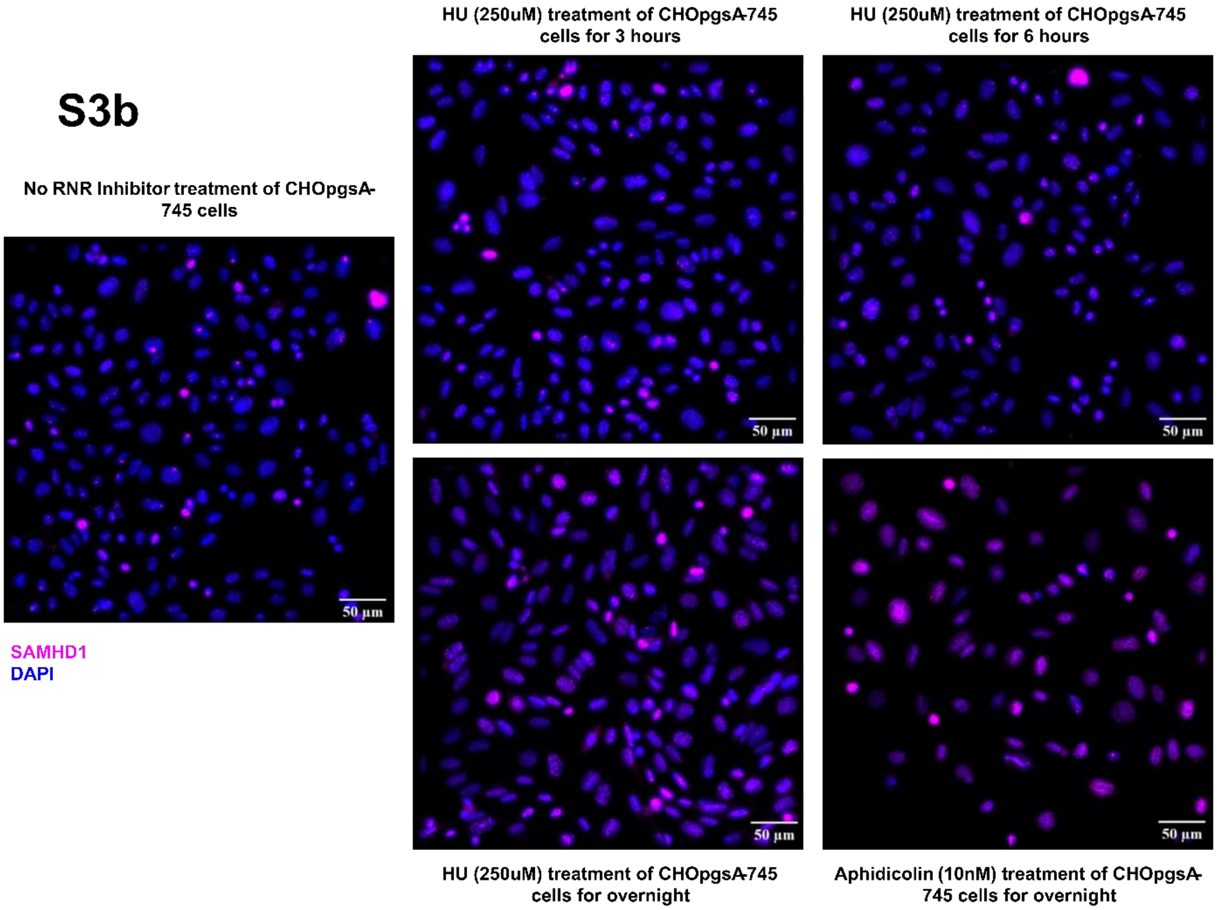
HU (250uM) treatment of CHOpgsA-745 cells for 3 hours
HU (250uM) treatment of CHOpgsA-745 cells for 6 hours
No RNR Inhibitor treatment of CHOpgsA-745 cells
50 µm
50 µm
50 µm
50 µm
50 µm
HU (250uM) treatment of CHOpgsA-745 cells for overnight
Aphidicolin (10nM) treatment of CHOpgsA-745 cells for overnight
SAMHD1
DAPI

Supplemental Figure S3. SAMHD1 Present in the cells under this study

S3a) SAMHD1 staining was performed with a 1:500 dilution of anti-SAMHD1 antibody (ab128107), and SAMHD1 expression was detected using an anti-mouse Donkey Rhodamine Red™-X (RRX) AffiniPure Fab Fragment (715-297-003) in cell lines used in this study (CHOpgsA-745, OMK, and TZMbl). Controls were SAMHD1 staining secondary antibody control and SAMHD1 staining in THP-1 (unactivated).

S3b) CHOpgsA-745 cells treated with HU (250 µM) for 3 hours, 6 hours and overnight followed by SAMHD1 staining. Controls were CHOpgsA-745 cells received no HU treatment and treated with Aphidicolin (10 nM) overnight.

The displayed images represent at least three independent biological repeats. Scale bar: 10, 15, 50 µm.

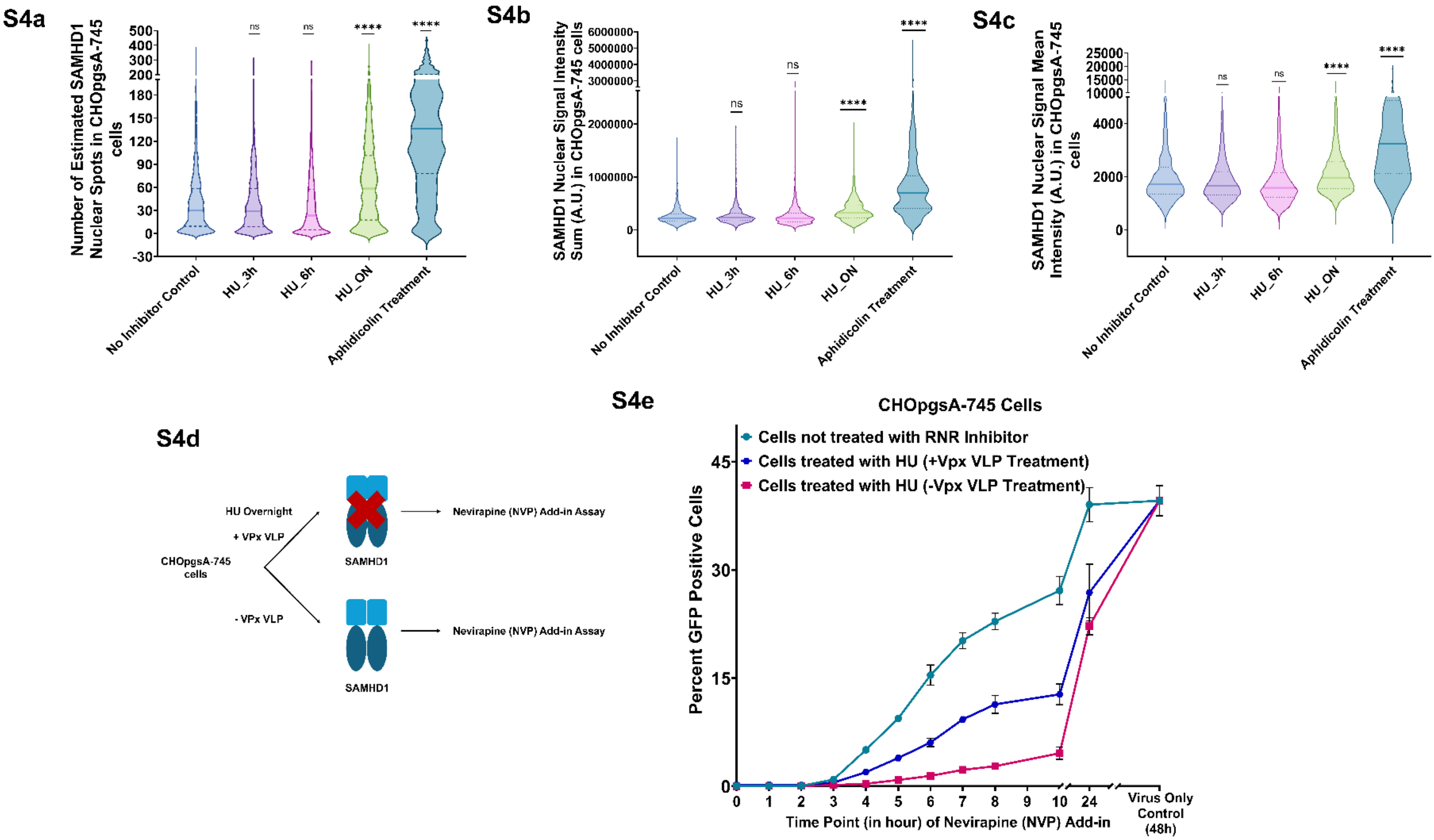

Supplemental Figure S4. Redistribution of Endogenous SAMHD1 after RNR inhibition.

S4a, S4b, S4c) CHOpgsA-745 cells treated with HU (250 µM) at different time-points followed by SAMHD1 staining. Redistribution dynamics of endogenous SAMHD1 in response to blockade of *de novo* pathway of dNTP biosynthesis was accessed by quantification of SAMHD1 nuclear spots, SAMHD1 nuclear signal intensity sum, SAMHD1 nuclear signal mean intensity and compared with no HU treatment control. Other conditions were CHOpgsA-745 cells treated with Aphidicolin (10nM) overnight. Per conditions around 1500 to 2000 cells were analyzed. Statistical significance was determined using one-way ANOVA with Dunnett's post-hoc correction to compare each treatment group to the no inhibitor control.

S4c) Experimental layout to access the functional role of SAMHD1 endogenously redistributed in response to blockade of *de novo* pathway of dNTP biosynthesis.

S4d) CHOpgsA-745 cells treated with Vpx (SIVmac$_{251}$) VLP during the overnight HU (250 µM) treatment and the sensitivity to the timed addition of NVP (10 µM) treatment (HIV-1 Reverse Transcription Kinetics) was accessed and compared to cells that received no Vpx (SIVmac$_{251}$) VLP treatment. Control condition were cells received neither treatment. Data are representative of three independent biological experiments. Error bars indicate the standard deviation (SD) of three technical replicates within this representative experiment.

Significance levels are indicated as follows: **** p <0.0001, ns = non-significant.

Virus Preparation	p24 (ng/ml)
VSV-G pseudotyped HIV-1 dual-labeled (HIV-1: iGFP and mRuby3-Integrase) virus prep#1	88.24

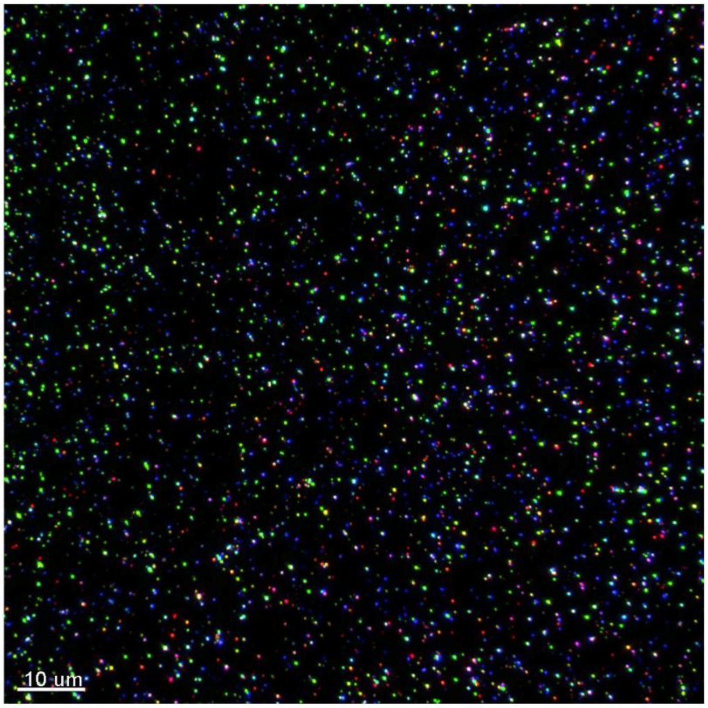

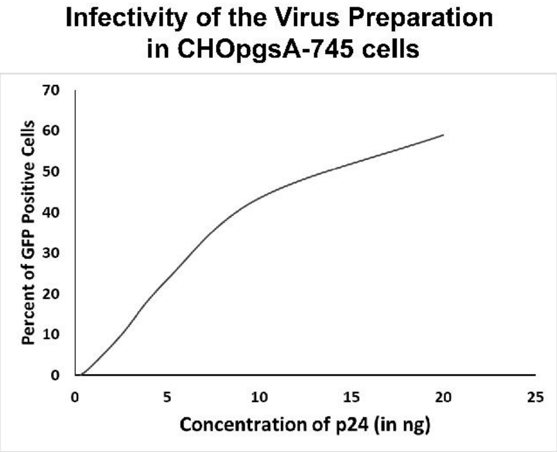

Virus Preparation	% of iGFP in mature CA
VSV-G pseudotyped HIV-1 dual-labeled (HIV-1: iGFP and mRuby3-Integrase) virus prep#1	56.34

Supplemental Figure S5. Characterization of VSV-G Pseudotyped HIV-1 dual labeled (HIV-1: iGFP and mRuby3-Integrase) virus preparation.

The VSV-G pseudotyped HIV-1 dual-labeled (HIV-1: iGFP and mRuby3-Integrase) virus preparation underwent thorough characterization, including quantification of p24 by ELISA, assessment of dual labeling efficiency via staining with Anti-HIV-1 p24 Recombinant Antibody (clone AG3) on coverslips and counting colocalized particles (GFP, Ruby3, and AF647 capturing Anti-HIV-1 p24 Recombinant Antibody directed against HIV-1 matured core), and determination of infectivity in CHOpgsA-745 cells by synchronized infection with different amounts of virus (normalized by p24 value) and quantification of percent GFP-positive cells. The described characterization represents one of several virus preparations. Scale bar: 10 µm.

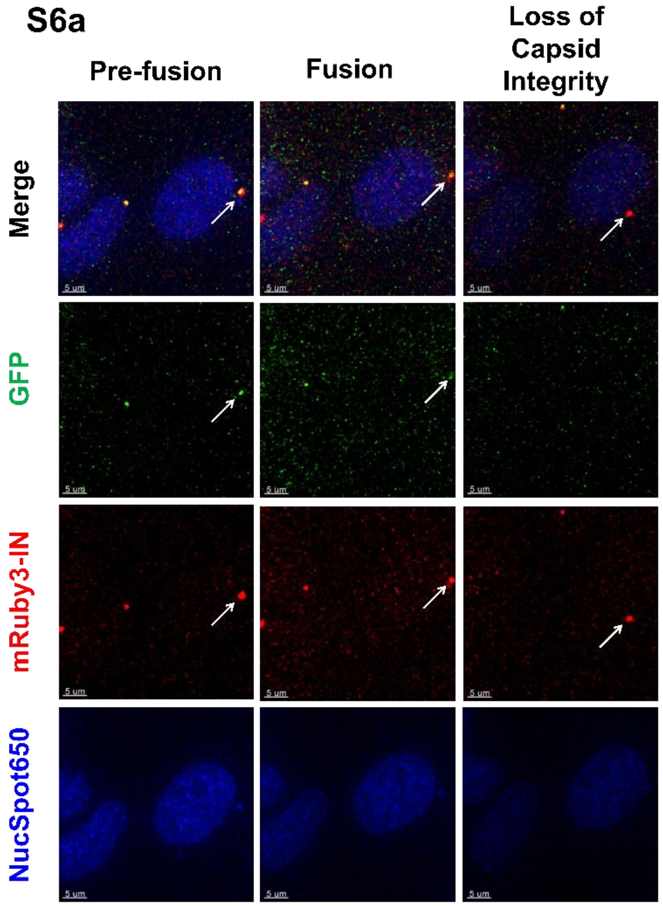

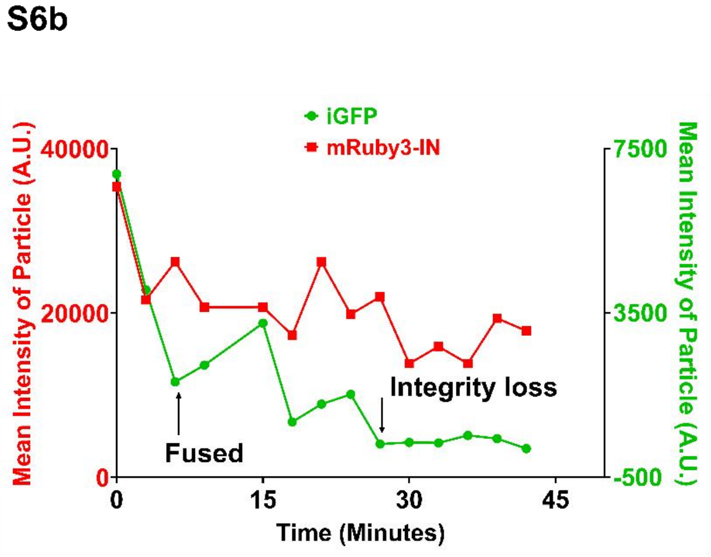

Supplemental Figure S6. HIV-1 Core Initiation of Uncoating in CHOpgsA-745 cells without any RNR inhibitor treatments.

S6a, S6b) Representative images from the capsid integrity assay in CHOpgsA-745 cells received no RNR inhibitor treatment (S6a, also Movie S1). Quantification of intensity over time corresponding to the images shown in panels S6a. The sharp decline in GFP signal denotes viral fusion events, while a complete loss of GFP signal associated with mRuby3-IN indicates the initiation of HIV-1 core uncoating. Arrow indicates the fluorescently labeled viral particle of interest across different channels. Scale bar: 5 µm. A similar loss of capsid integrity profile was reported previously by our laboratory (3).

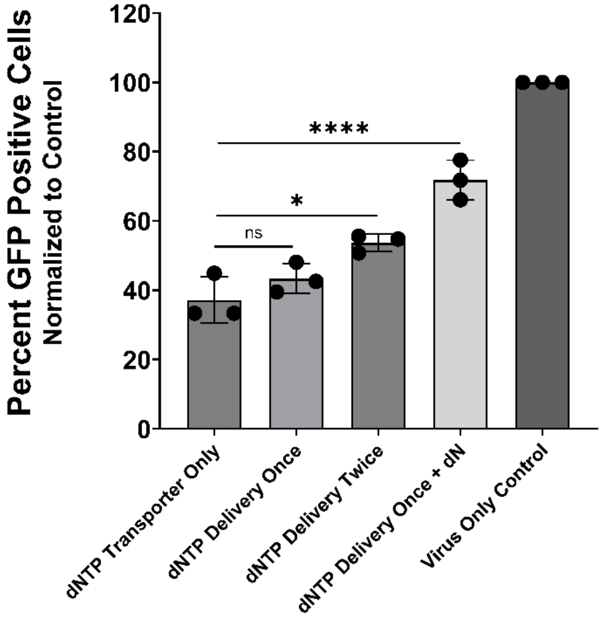

Supplemental Figure S7. Restoration of HIV-1 infectivity in HU-treated CHOpgsA-745 cells following external dNTP delivery.

This figure presents data from multiple biological repeats assessing HIV-1 infectivity restoration under RNR inhibition (HU, 1000 µM) in CHOpgsA-745 cells. Unlabeled dNTPs were delivered using the BioTracker™ NTP-Transporter, and infectivity was measured across independent experiments. These data complement the representative results shown in the main Figure 6a-b.

The bar plots shown are the percentage of GFP positive cells at each condition normalized by setting the percentage of GFP positive cells at virus only control as 100%. Statistical significance was determined using one-way ANOVA with Dunnett's post-hoc correction to compare each treatment group to the dNTP transporter delivery control. Error bars represent the standard error (SE) from three independent biological replicates. Significance levels are denoted as follows: **** $p < 0.0001$, * $p = 0.0130$, ns = not significant.

Supplemental Movie Legends

Movie S1: Time-lapse image of an NH_4Cl-synchronized VSV-G pseudotyped dual-labeled (iGFP and mRuby3-IN) HIV-1 particle within CHOpgsA-745 cells that received no RNR inhibitor treatment. The right box shows the dual drop of GFP signals in the particle anchored by a mRuby3-IN signal. Insets on the left show the respective florescent signal of the particle over time as labeled. Viral fusion is marked by a sharp GFP drop (~5 minutes), while the initiation of HIV-1 core uncoating is indicated by a complete loss of GFP (~ 38 minutes) signals associated with the tracked mRuby3-IN signal. A similar capsid integrity loss profile was previously reported by our laboratory (3). Nucleus was stained with NucSpot650 and is shown in blue.

Movie S2: Time-lapse image of an NH_4Cl-synchronized, VSV-G pseudotyped dual-labeled (iGFP and mRuby3-IN) HIV-1 particle within CHOpgsA-745 cells treated with HU. The right box shows the drop of GFP signals in the particle anchored by a mRuby3-IN signal. Insets at the bottom left show the respective florescent signal of the particle over time as labeled. Viral fusion is marked by a sharp GFP drop (~ 14 minutes), while fraction of GFP signal remains in the intact core associated with mRuby3-IN signal until 120 minutes. The top-left plot presents background-adjusted mean intensity of GFP and mRuby3-IN signals over time. Nucleus was stained with NucSpot650 and is shown in blue.

Movie S3: Time-lapse image of an NH_4Cl-synchronized, VSV-G pseudotyped dual-labeled (iGFP and mRuby3-IN) HIV-1 particle within CHOpgsA-745 cells treated with COH29. The right box shows the drop of GFP signals in the particle anchored by a mRuby3-IN signal. Insets at the bottom left show the respective florescent signal of the particle over time as labeled. Viral fusion is marked by a sharp GFP drop (~ 5 minutes), while fraction of GFP signal remains in the intact core associated with mRuby3-IN signal until 120 minutes. The top-left plot presents background-adjusted mean intensity of GFP and mRuby3-IN signals over time. Nucleus was stained with NucSpot650 and is shown in blue.

Movie S4: Time-lapse image of an NH_4Cl-synchronized, VSV-G pseudotyped dual-labeled (iGFP and mRuby3-IN) HIV-1 particle within CHOpgsA-745 cells treated with HU, followed by external unlabeled dNTP delivery starting at 120 minutes. The right box shows the dual drop of GFP signals in the particle anchored by a mRuby3-IN signal. Insets at the bottom left show the respective florescent signal of the particle over time as labeled. Viral fusion is marked by a sharp GFP drop (~ 5 minutes), while fraction of GFP signal remains in the intact core associated with mRuby3-IN signal until 120 minutes. The unlabeled dNTPs delivery process took around 50 minutes followed by resuming the time-lapsed imaging. The immediate next time frame after dNTP delivery process, a complete loss of GFP (around 171 minutes) signals associated with the tracked mRuby3-IN signal was observed. The top-left plot presents background-adjusted mean intensity of GFP and mRuby3-IN signals over time. Nucleus was stained with NucSpot650 and is shown in blue.

Reference

1. Pachitariu M, Stringer C. 2022. Cellpose 2.0: how to train your own model. Nature methods 19:1634-1641.
2. Bankhead P, Loughrey MB, Fernández JA, Dombrowski Y, McArt DG, Dunne PD, McQuaid S, Gray RT, Murray LJ, Coleman HG. 2017. QuPath: Open source software for digital pathology image analysis. Scientific reports 7:1-7.
3. Mamede JI, Cianci GC, Anderson MR, Hope TJ. 2017. Early cytoplasmic uncoating is associated with infectivity of HIV-1. Proceedings of the National Academy of Sciences 114:E7169-E7178.
